# Age-related and dual task-induced gait alterations and asymmetry: optimizing the Semmelweis Study gait assessment protocol

**DOI:** 10.1007/s11357-025-01722-6

**Published:** 2025-06-06

**Authors:** Peter Mukli, Mihaly Muranyi, Ágnes Lipecz, Zsofia Szarvas, Tamás Csípő, Anna Ungvari, Mónika Fekete, Vince Fazekas-Pongor, Anna Peterfi, Ágnes Fehér, Norbert Dosa, Noémi Mózes, Csilla Kaposvári, Anna Aliquander, Wei Yi Hung, Dávid Major, Zalan Kaposzta, Attila Matiscsák, Gabriella Dörnyei, Zoltan Benyo, Adam Nyul-Toth, Roland Patai, Boglarka Csik, Rafal Gulej, Attila Kallai, Marton Sandor, Peter Varga, Adam G. Tabak, Stefano Tarantini, Róza Ádány, Béla Merkely, Anna Csiszar, Andriy Yabluchanskiy, Zoltan Ungvari

**Affiliations:** 1 Vascular Cognitive Impairment, Neurodegeneration and Healthy Brain Aging Program, Department of Neurosurgery, University of Oklahoma Health Sciences Center, Oklahoma City, OK USA; 2 Oklahoma Center for Geroscience and Healthy Brain Aging, University of Oklahoma Health Sciences Center, Oklahoma City, OK USA; 3 International Training Program in Geroscience, Doctoral College, Health Sciences Division/Institute of Preventive Medicine and Public Health, Semmelweis University, Budapest, Hungary; 4 Doctoral College, Health Sciences Division, Semmelweis University, Budapest, Hungary; 5 Institute of Preventive Medicine and Public Health, Semmelweis University, Budapest, Hungary; 6 Fodor Center for Prevention and Healthy Aging, Semmelweis University, Budapest, Hungary; 7 Faculty of Health Sciences, Semmelweis University, Budapest, Hungary; 8 Institute of Translational Medicine, Semmelweis University, 1094 Budapest, Hungary; 9 Cerebrovascular and Neurocognitive Disorders Research Group, HUN-REN, Semmelweis University, 1094 Budapest, Hungary; 10 UCL Brain Sciences, University College London, London, UK; 11 Department of Internal Medicine and Oncology, Faculty of Medicine, Semmelweis University, Budapest, Hungary; 12 Department of Health Promotion Sciences, College of Public Health, University of Oklahoma Health Sciences Center, Oklahoma City, OK USA; 13 The Peggy and Charles Stephenson Cancer Center, University of Oklahoma Health Sciences Center, Oklahoma City, OK USA; 14 Department of Public Health and Epidemiology, Faculty of Medicine, University of Debrecen, Debrecen, Hungary; 15 HUN-REN-UD Public Health Research Group, Department of Public Health and Epidemiology, Faculty of Medicine, University of Debrecen, Debrecen, Hungary; 16 National Laboratory for Health Security, Center for Epidemiology and Surveillance, Semmelweis University, Budapest, Hungary; 17 Heart and Vascular Center, Semmelweis University, Budapest, Hungary

**Keywords:** Gait asymmetry, Aging, Dual-task walking, Cognitive load, Gait variability, Semmelweis Study, Mobility decline

## Abstract

Gait alterations are recognized as early markers of age-related decline and cognitive impairment. Dual-task assessments, which impose cognitive load while walking, provide valuable insights into gait control limitations and cognitive-motor interactions in aging populations. This study evaluates age-related and cognitive load-induced changes in gait parameters, with a particular focus on asymmetry, and aims to optimize the gait assessment protocol for the Semmelweis Study framework. The Semmelweis Study is a large-scale workplace cohort investigating the determinants of unhealthy aging and promoting healthy brain aging by identifying risk factors and protective mechanisms influencing vascular, metabolic, and neurocognitive decline. As part of this initiative, gait analysis is emerging as a critical tool for assessing functional aging, detecting early signs of mobility and cognitive impairment, and contributing to biological age assessment. A cross-sectional analysis was conducted on adults aged 23 to 87 years using a pressure-sensitive walkway system. Participants were evaluated under single-task conditions (normal walking) and dual-task conditions (walking while performing a concurrent cognitive task). Spatiotemporal gait parameters, asymmetry indices, and dual-task costs were analyzed to assess age-related changes in gait performance and cognitive-motor interactions. Aging was associated with significant reductions in gait speed, step length, and stride length, along with a corresponding increase in gait asymmetry. Dual-task conditions exacerbated these alterations, indicating age-related impairments in cognitive-motor integration. Asymmetry indices were sensitive to aging effects, suggesting their potential as biomarkers for functional decline. The dual-task cost on gait was significantly greater in older adults, reinforcing the interplay between cognitive and motor systems in aging. Age-related gait alterations, particularly under cognitive load, underscore the importance of comprehensive gait assessments in aging research. Our findings contribute to the optimization of the Semmelweis Study gait assessment protocol by identifying key gait parameters that capture functional decline and biological aging. Integrating dual-task gait analysis into large-scale epidemiological studies has the potential to enhance early detection of brain health decline, refine biological age estimation, and guide targeted interventions to support healthy aging and neuromotor resilience.

## Introduction

The aging of populations presents an urgent public health challenge across the European Union (EU) and beyond [1]. Currently, more than 100 million individuals aged 65 and older reside in the EU and the UK, with this number projected to increase to nearly 150 million by 2050. This demographic shift—representing over 21% of the EU population as of 2025, with an expected rise to 29.5% by 2050 [1, 2]—underlines the critical need to address age-associated diseases, mobility decline, and cognitive impairment to maintain independence among older adults. As the elderly population grows, the strain on social, healthcare, and economic resources will intensify, necessitating comprehensive strategies to promote healthy aging and extend health span in addition to lifespan.

Safeguarding the health of aging populations is also integral to achieving the Sustainable Development Goals (SDGs), ensuring that increased longevity is accompanied by an improved quality of life. However, disparities in health outcomes across EU member states pose significant challenges to aging-related public health efforts. For example, Hungary faces unique demographic pressures, with projections indicating that the percentage of its population aged 65 and older will rise from 20.3% in 2021 to 27.9% by 2050 [3, 4]. The interplay between population aging, public health, labor market sustainability, and social services highlights the urgent need for a thorough understanding of aging-related health risks and the factors contributing to successful aging.

In response to these challenges, Semmelweis University in Budapest, Hungary, established the Fodor Center for Prevention and Healthy Aging and launched a comprehensive initiative to promote healthy aging and drive healthcare reform [5]. As Hungary’s largest healthcare provider and a leading health sciences institution in Central Europe, Semmelweis University leverages its resources to tackle the complexities of aging through three key components: (1) The Semmelweis Study—A longitudinal workplace cohort study investigating the determinants of unhealthy aging [6]. (2) The Semmelweis Workplace Health Promotion Model Program—A structured initiative to promote healthy aging in the workplace setting. (3) and a pioneering multidisciplinary program in preventive medicine focused on primary healthcare reform and early intervention [5]. The Semmelweis Study is a prospective cohort study targeting all university employees and faculty aged 25 years and older, encompassing over 12,000 participants from diverse occupational and socioeconomic backgrounds [6]. This study provides a unique opportunity to assess lifestyle, environmental, and occupational factors contributing to unhealthy aging and the genesis and progression of age-related chronic diseases. A major focus is to identify vascular and metabolic risk factors for cognitive decline, integrating high-throughput methodologies to assess brain health, mobility, and functional aging [6].

Gait, once considered a simple motor activity, is now recognized as a complex neuromotor process influenced by vascular health, cognitive function, and musculoskeletal integrity [7–12]. Emerging evidence suggests that gait characteristics can serve as early indicators of cognitive decline, neurovascular dysfunction [13–20], and frailty, making gait analysis a powerful tool for aging research [15, 20–24]. Among gait parameters, gait asymmetry [25–31] and variability [18, 20, 32–38] have been identified as potential biomarkers of neurocognitive impairment, with greater asymmetry associated with an increased risk of cognitive and functional decline. Symmetry in gait is an important measure in aging studies, as asymmetry may reflect underlying neuropathologies, such as stokes, cerebral microhemorrhages, lacunar strokes, and white matter hyperintensities, which disproportionately affect one side of the brain [26, 27, 39, 40].

Dual-task gait assessments, which impose cognitive load while walking, provide valuable insights into cognitive-motor interactions and serve as a sensitive measure of brain aging [41–43]. Older adults often exhibit a greater dual-task cost, characterized by slower gait speed among others, reflecting underlying deficits in executive function and neural compensation mechanisms [41, 42, 44]. These gait alterations are associated with an elevated risk of falls, frailty [25, 43, 45–49], and cognitive impairment, underscoring the need for high-throughput, reproducible gait assessments in large-scale epidemiological studies.

As part of the Semmelweis Study, this pilot investigation focuses on age-related and cognitive load-induced gait alterations, with a particular emphasis on gait asymmetry as a potential marker of functional and cognitive aging. By analyzing gait parameters in both single-task and dual-task conditions, this study aims to optimize the gait assessment protocol for future large-scale analyses within the Semmelweis Study framework. These findings will contribute to refining methodological frameworks for gait assessments in longitudinal studies, enhancing the early detection of mobility impairments and cognitive decline, and supporting the development of interventions to promote cognitive and neuromotor resilience in aging populations. By integrating gait assessment into the broader context of brain aging and public health, this study provides critical insights into the determinants of functional decline and helps shape future interventions for healthy aging.

## Materials and methods

### Study participants

This cross-sectional study recruited 103 adults spanning a wide age range (23–87 years; 45 males). Based on widely used cut-off values, participants were categorized into two age groups. The Younger Adults (YA) group included individuals under 65 years of age (*n* = 62, 27 males, 35 females) and encompassed both young and middle-aged adults, divided at the 45-year threshold. The Older Adults (OA) group consisted of participants aged 65 years and older (*n* = 41, 18 males, 23 females). All participants underwent medical history screenings prior to enrollment to determine eligibility. Inclusion criteria required participants to be able to read and write, possess adequate hearing and vision for the assessments, and be competent to provide informed consent. Exclusion criteria included conditions that could directly impact spatiotemporal gait patterns, such as neurological disorders (e.g., Parkinson’s disease, multiple sclerosis); musculoskeletal conditions (history of orthopedic surgeries or musculoskeletal diseases); and cardiovascular or metabolic conditions. To minimize external influences on gait performance, participants were instructed to abstain from caffeine for at least 6 h prior to testing and ensure a minimum of 7 h of sleep before functional assessments. Informed consent was obtained from all participants before enrollment. The study’s procedures and protocols were approved by the institutional review boards of the participating institutions: the Hungarian Medical Research Council (approval number #53,981–2/2023/809) and the Institutional Review Board of the University of Oklahoma Health Sciences Center (approval numbers #8129 and #9555).

### Measurement protocol

Gait data were acquired using a 20-ft-long pressure-sensitive electronic mat (ProtoKinetics Zeno™ Walkway Gait Analysis System, Havertown, PA, USA), which captures both spatial (distance) and temporal (time-based) parameters of gait as previously described [20]. The assessment protocol consisted of single-task and cognitive dual-task conditions, with participants walking at their self-selected speed, wearing their regular shoes, and moving freely without external constraints.

The dual-task condition was designed to evaluate the effect of increased cognitive load on gait patterns, reflecting the limited attentional resources available during simultaneous cognitive engagement. In the single-task condition, participants completed five walking trials, covering ten passes across the gait mat, turning at each end. Following this, the dual-task condition was introduced, during which participants performed serial subtraction while walking. Specifically, they were instructed to subtract 7 repeatedly from 500, say each number aloud, and continue the task even if they recognized an earlier mistake. This approach ensured continuous cognitive engagement while walking, allowing for the examination of two distinct gait patterns within the same participant.

### Assessment of gait patterns

Table 1 provides an overview of the gait parameters captured by the ProtoKinetics Gait Analysis System, derived from raw pressure maps. A comprehensive characterization of gait patterns includes spatial and temporal profiles as well as key parameters reflecting balance control across different phases of the gait cycle.

**Table 1:** Parameters of gait pattern captured by the pressure-sensitive gait mat

Gait parameter name (abbreviation)	Definition (measurement unit)
*Gait speed*	The covered distance during a given walking trial divided by time
*Cadence*	The number of steps during a given walking trial divided by time
*Step time (SteTim)*	It refers to the duration of a single step, measured as the time interval between the initial contact of one foot and the initial contact of the opposite foot (sec)
*Stride time (StrTim)*	A key parameter capturing the duration of gait cycle: the period of time from first contact of one foot, to the following first contact of the same foot (sec)
*Step length (SteLen)*	Step length is the distance between corresponding successive heel points of opposite feet, measured parallel to the direction of progression for the ipsilateral stride (cm)
*Stride length (StrLen)*	The distance from the heel of one foot to the following heel of the same foot (cm)
*Stride width (StrW)*	Stride width is the perpendicular distance between the line connecting the two ipsilateral foot heel contacts (stride) with the contralateral heel contact between those events (cm)
*Stance*	The stance phase begins when the foot first touches the ground and ends when the same foot leaves the ground. This measure is expressed as a percentage of gait cycle time spent in the stance phase and averaged between left and right foot (%)
*Swing*	The period of time when the foot is not in contact with the ground. It is presented as a percentage of the gait cycle time (%)
*Single support (SSup)*	The percentage of gait cycle time when only the current foot is in contact with the ground (%)
*Total double support (TDSup)*	The percentage of gait cycle time when both feet are in contact with the ground during stance phase (%)
*Integrated pressure (IntP)*	The area under the footfall pressure curve during ground contact for the given footfall
*Stance center of pressure distance (StCOPd)*	It represents the center of pressure (COP) start to end distance as a percent of the maximum foot length
*Single support center of pressure distance (SSCOPd)*	It represents the COP start to end distance in single support as a percent of the maximum foot length

Temporal parameters include gait speed, cadence, step time, and stride time, which provide insights into walking efficiency and rhythm. Spatial parameters such as step length, stride length, and stride width describe the overall geometry of movement. The relative durations of gait cycle phases are assessed through stance, single support, and dual support times, offering critical information about weight distribution and stability during walking.

Additionally, parameters reflecting balance control include integrated pressure, stance center of pressure (COP) distance, and single support center of pressure distance. These measures provide insights into postural stability and neuromotor control, offering a more detailed evaluation of balance across different gait phases.

### Data analysis

The evaluation of gait began with an initial review of the raw data, during which incomplete steps and artefactual patterns were identified and removed if confirmed. Following data cleaning, individual-level analyses were conducted by computing the arithmetic mean for each gait parameter listed in Table 1. Additionally, two measures of asymmetry were derived for each parameter to assess limb-to-limb differences in gait patterns.

Asymmetry was quantified using the Ratio Index (RI), which was calculated as the ratio of values (X) for the two limbs per stride, following Eq. 1. This ratio was then averaged across the entire walking trial to obtain a representative measure of gait asymmetry.

1 $$RI=100\bullet \left|1-exp\left(-\left|\text{ln}\left(\frac{{X}_{\text{right}}}{{X}_{\text{left}}}\right)\right|\right)\right|\left[\%\right]$$

Perfect symmetry is indicated by an RI value of 0, reflecting equal limb contributions to gait. In contrast, an RI value of 100 corresponds to the highest possible asymmetry, theoretically representing a complete imbalance between the two limbs. This measure provides a standardized approach to quantifying gait asymmetry, allowing for meaningful comparisons across individuals and conditions [50].

For the Symmetry Index (SI), the difference between the values corresponding to the two limbs is normalized to their average within the same stride, following Eq. 2. This normalization accounts for individual variability in gait parameters and provides a scale-independent measure of asymmetry, facilitating comparisons across participants and conditions.

2 $$SI=100\bullet \frac{\left|{X}_{\text{left}}{-X}_{\text{right}}\right|}{0.5\bullet \left({X}_{\text{left}}{+X}_{\text{right}}\right)}\left[\%\right]$$

Similar to the Ratio Index, an SI value of 0 indicates perfect left–right symmetry for a given gait parameter, reflecting equal contributions from both limbs. Conversely, an SI value of 100% represents maximal asymmetry. This standardized measure allows for quantitative assessment of gait balance and enhances comparability across individuals and conditions.

Both symmetry parameters, RI and SI, are expressed as percentages and take a value of 0 when ${X}_{\text{left}}{=X}_{\text{right}}$, indicating perfect symmetry. While in theory, RI = 100% or SI = 100% corresponds to maximal asymmetry, these values are can only be approximated on empirical data. Extreme values occur primarily when *X* is an outlier, such as when one limb’s value is ≈0 or exceeds the contralateral value by several magnitudes, which is usually due to improperly captured steps rather than true physiological asymmetry.

### Statistical tests

The normality of data distributions was assessed using the Lilliefors test. As the majority of the samples were not normally distributed, data are presented as median (IQR), unless stated otherwise. Given the non-normal distribution, rank correlation analyses were used to examine the relationship between gait parameters and chronological age, treating all variables as continuous. Spearman correlation coefficients were calculated separately for single-task and dual-task conditions, as well as for dual-task cost, which was defined as the arithmetic difference between gait parameters recorded under dual-task and single-task conditions.

After defining age categories, gait parameters were compared between younger and older adults using the Mann–Whitney test, except for parameters where both groups exhibited normal distributions, in which case an unpaired two-tailed *t*-test was applied. Levene’s test was used to assess variance homogeneity, and Welch’s correction was applied when variances were unequal. The effect of dual-task conditions on gait parameters was evaluated using the Wilcoxon test, unless normality assumptions required for a paired *t*-test were met. The level of significance was set at *α* = 0.05.

## Results

### Participants characteristics

Table 2 provides an overview of the sex distributions of the study population, along with participants’ educational background, active medical conditions, and medication use. Baseline physiological data, including relevant health parameters, are summarized in Table 3. These demographic and health characteristics offer important context for interpreting gait performance across age groups and cognitive load conditions.

**Table 2:** Demographics data

*Characteristics*	**Young (*n* = 62)**		**Elderly (*n* = 41)**	
	***n***	**%**	***n***	**%**
*Sex*				
*Male*	27	26.2	18	17.5
*Female*	35	34.0	23	22.3
*Highest level of education*				
*Academic doctorate degree*	17	16.5	5	4.8
*Professional doctorate degree*	11	10.7	0	0
*Master degree*	20	19.4	11	10.7
*Bachelor degree*	11	10.7	15	14.6
*Other, no college or university degree*	3	2.9	10	9.7
*Handedness*				
*Left*	7	6.8	4	3.9
*Right*	55	53.4	37	35.9

**Table 3:** Health conditions, medications, and baseline physiological parameters of the study population

*Diseases, medications and baseline physiological measures*	**Young (*n* = 62)**		**Elderly (*n* = 41)**	
	***n***	**%**	***n***	**%**
Active diseases*				
*Hypertension (controlled)*	5	4.9	23	22.3
*Other cardiovascular diseases*	3	2.9	3	2.9
*Diabetes mellitus (controlled)*	2	1.9	5	4.9
*Hypothyroidism*	1	1.0	2	2.0
*Other metabolic disorder*	2	1.9	6	17.6
*Psychiatric disease*	3	2.9	7	6.8
*Neurological disease*	2	1.9	3	2.9
*Diseases of bones, joints and muscle*	7	3.9	13	12.5
*Other medical conditions*	7	6.8	12	11.7
Current smoker**	6	5.8	6	5.8
Medications***				
*AT1-receptor blocker*	1	1.0	6	5.8
*ACE-inhibitor*	1	1.0	7	6.8
*β-blocker*	1	1.0	7	6.8
*Ca-antagonist*	2	1.9	7	6.8
*Diuretic*	3	2.9	10	9.7
*Statin*	2	1.9	12	11.7
*Estrogen supplement*	1	1.0	6	3.9
*Thyroid supplement*	1	1.0	11	10.7
*Other prescribed drugs*	22	21.4	26	25.2
*Baseline data*	**Mean**	**S.D**	**Mean**	**S.D**
Systolic blood pressure (mmHg)*	116.4	12.8	126.3	16.2
*Diastolic blood pressure (mmHg)*	77.6	10.3	74.9	8.5
Mean arterial blood pressure (mmHg)*	90.5	10.7	92.1	8.6
*Heart rate (1/min)*	66.0	9.8	64.1	13.1
*Body mass index (BMI; kg/m*^*2*^)*	25.3	5.3	26.6	4.3

### Basic statistics of gait parameters

To evaluate age-related changes in fundamental gait characteristics, rank correlation analyses were performed between chronological age and the arithmetic mean of gait parameters for each walking session of each individual. Additionally, gait parameters were compared between younger and older adults, with non-parametric descriptive statistics reported in Table 4.

**Table 4:** Age-stratified gait metrics including median, ratio index (RI), and symmetry index (SI) for single- and dual-task conditions

Gait parameter median [IQR]	Basic statistics (mean)				Ratio index				Symmetry index			
	**Single task**		**Dual task**		**Single task**		**Dual task**		**Single task**		**Dual task**	
	**Young**	**Aged**	**Young**	**Aged**	**Young**	**Aged**	**Young**	**Aged**	**Young**	**Aged**	**Young**	**Aged**
*Gait speed (cm/s)*	117.88 [29.98]	106.11 [22.95]	106.78 [22.22]	90.03 [31.39]								
*Cadence (steps/min)*	105.04 [9.66]	106.14 [14.80]	100.11 [11.36]	98.65 [17.31]								
*Step time (s)*	0.570 [0.048]	0.565 [0.076]	0.600 [0.065]	0.606 [0.111]	1.268 [1.778]	1.6043 [1.770]	1.312 [1.852]	1.879 [2.402]	0.8000 [1.500]	0.7807 [0.714]	0.7220 [1.049]	0.7701 [1.168]
*Stride time (s)*	1.141 [0.089]	1.131 [0.155]	1.195 [0.125]	1.211 [0.220]	0.3233 [0.564]	0.2730 [0.383]	0.4150 [0.711]	0.4218 [1.069]	0.3416 [0.387]	0.3393 [0.392]	0.1963 [0.362]	0.3441 [0.518]
*Step length (cm)*	67.158 [8.562]	58.590 [10.33]	64.14 [10.46]	54.119 [12.39]	1.7078 [2.773]	3.0827 [3.572]	2.0549 [2.182]	3.0693 [5.230]	0.7856 [1.383]	1.7376 [2.357]	0.9671 [1.374]	1.6725 [3.137]
*Stride length (cm)*	134.34 [17.22]	116.93 [21.67]	128.44 [20.84]	107.90 [25.20]	0.2742 [0.425]	0.3012 [0.450]	0.3560 [0.451]	0.4653 [0.655]	0.1498 [0.255]	0.1815 [0.321]	0.2333 [0.291]	0.2458 [0.226]
*Stride width (m)*	7.668 [3.561]	7.1435 [4.738]	8.004 [2.981]	8.4675 [4.258]	2.6553 [3.353]	2.8238 [3.632]	1.978 [4.475]	1.7589 [2.580]	1.800 [2.673]	1.256 [3.096]	1.5711 [2.918]	1.6726 [2.697]
*Stance (%)*	64.280 [2.537]	64.650 [2.749]	64.519 [2.376]	65.902 [3.896]	0.9967 [1.215]	1.3581 [1.698]	0.8978 [1.072]	1.4324 [1.609]	0.4444 [0.604]	0.5676 [0.798]	0.5219 [0.478]	0.8085 [0.851]
*Swing (%)*	35.72 [2.537]	35.351 [2.749]	35.481 [2.376]	34.099 [3.896]	1.7481 [2.127]	2.3146 [3.130]	1.6722 [1.941]	2.9012 [2.936]	0.7982 [1.092]	1.0100 [1.536]	0.9825 [0.864]	1.5264 [1.699]
*Single support (%)*	35.648 [2.362]	35.279 [2.619]	35.173 [2.568]	34.117 [2.688]	1.5209 [1.581]	2.1849 [3.611]	1.8816 [1.784]	2.2534 [3.007]	0.6984 [1.190]	1.1007 [1.738]	1.0103 [0.969]	1.4348 [1.569]
*Total double support (%)*	28.336 [4.670]	29.340 [5.400]	29.197 [4.685]	31.788 [6.516]	0.2797 [0.380]	0.3512 [0.505]	0.4053 [0.527]	0.4752 [0.808]	0.2984 [0.357]	0.3432 [0.397]	0.2374 [0.386]	0.2797 [0.389]
*Integrated pressure (Pa)*	145.93 [51.59]	167.11 [66.06]	158.53 [56.72]	177.00 [63.90]	2.1592 [2.632]	2.4357 [2.675]	2.3177 [2.485]	3.5939 [3.386]	0.8019 [0.925]	1.3341 [1.467]	0.9366 [1.139]	1.5089 [1.859]
*Stance COP distance (%)*	83.264 [3.899]	82.952 [3.352	82.982 [3.601]	82.908 [4.588]	2.6852 [4.038]	2.4514 [3.521]	2.0914 [2.375]	2.5335 [3.555]	1.0758 [1.604]	1.1736 [1.255]	0.9082 [1.321]	1.0346 [1.589]
*Single support COP distance*	39.325 [6.110]	34.688 [8.133]	37.411 [5.858]	31.761 [6.697]	1.5209 [1.581]	2.1849 [3.611]	1.8816 [1.784]	2.2534 [3.007]	1.7975 [2.113]	2.1064 [3.818]	2.2117 [2.294]	3.0112 [4.110]

### Temporal gait parameters

**Fig. 1:**
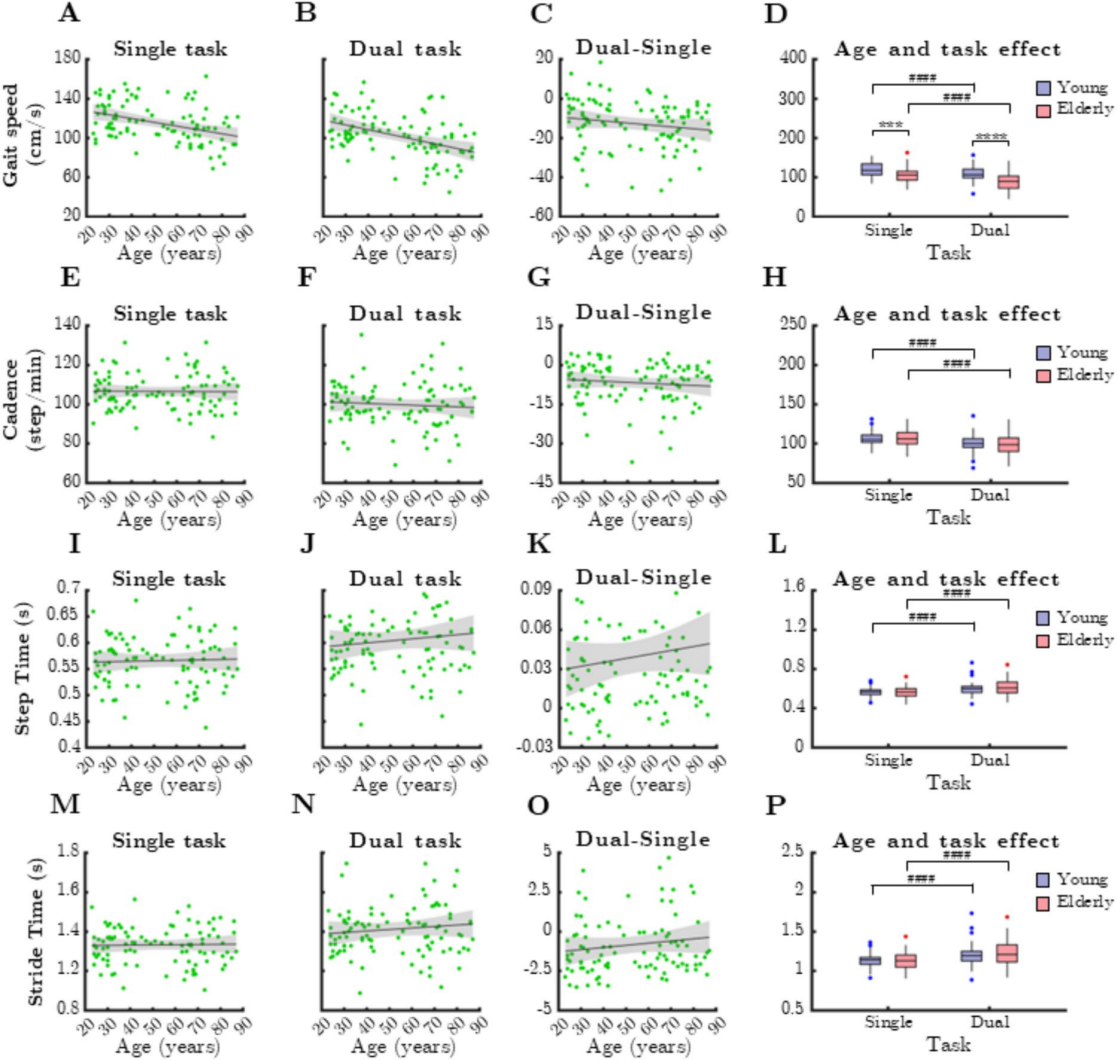
Relationship between age and temporal parameters of gait during single and dual task. SteTim: step time, StrTim: stride time. Black lines and grey shaded areas on the scatterplot represent the trendline and its 95% confidence interval, respectively. Box-whisker plots show data for the young (*n* = 60) and elderly (*n* = 41) groups separately in light purple-blue and light pink-red, respectively. Blue and red dots denote statistical outliers. **A** Gait speed during single task significantly decreases with age (Spearman’s *p* < 0.05). **B** Gait speed during dual task significantly decreases with age (Spearman’s *p* < 0.05). **C** Difference in gait speed between single and dual task, as a function of age. **D** Impact of age group and task condition on the median of gait speed. **E** Age-related changes in cadence, single task. **F** Age-related changes in cadence, dual task. **G** Difference in cadence between single and dual task, as a function of age. **H** Impact of age group and task condition on the median of cadence. **I** Age-related changes in mean SteTim, single task. **J** Mean SteTim corresponding to dual task significantly increases with age (Spearman’s *p* < 0.05). **K** Difference in mean SteTim between single and dual task, as a function of age. **L** Impact of age group and task condition on the median of mean SteTim. **M** Age-related changes in mean StrTim, single task. **N** Age-related changes in mean StrTim, dual task. **O** Difference in mean StrTim between single and dual task, as a function of age. **P** Impact of age group and task condition on the median of mean StrTim. Panels **D**, **H**, **L** and **P**: ^***^p < 0.001: age effect (Mann–Whitney test); ^***^: *p* < 0.001: age effect (Mann–Whitney test); ^****^: *p* < 0.0001: age effect (Mann–Whitney test); ^##^: *p* < 0.01, task effect (paired t-test); ^####^: *p* < 0.0001, task effect (Wilcoxon-test). On panels **A**–**C**, **E**–**G**, **I**–**K**, **M**–**O** a linear trendline ± [range] is plotted to capture linear associations

Among temporal gait parameters (Fig. 1), gait speed exhibited the strongest negative correlation with age under both single-task (Spearman’s rho =  − 0.3854, *p* < 0.0001) and dual-task conditions (rho =  − 0.4476, *p* < 0.0001). As expected, gait speed was significantly lower during dual-task walking (Wilcoxon test, *p* < 0.0001) (Fig. 1D). Older adults walked at a significantly slower pace in both conditions compared to younger adults (*p* < 0.0001, unpaired *t*-test with Welch’s correction).

No significant correlations were found between chronological age and other temporal gait variables, including cadence (Fig. 1E, F), step time (Fig. 1I, J), and stride time (Fig. 1M, N). Likewise, dual-task cost of temporal gait parameters did not correlate with age (Spearman’s *p* > 0.05). However, dual-task conditions significantly altered all temporal gait parameters in both age groups (*p* < 0.0001, Wilcoxon test).

### Spatial gait parameters

**Fig. 2:**
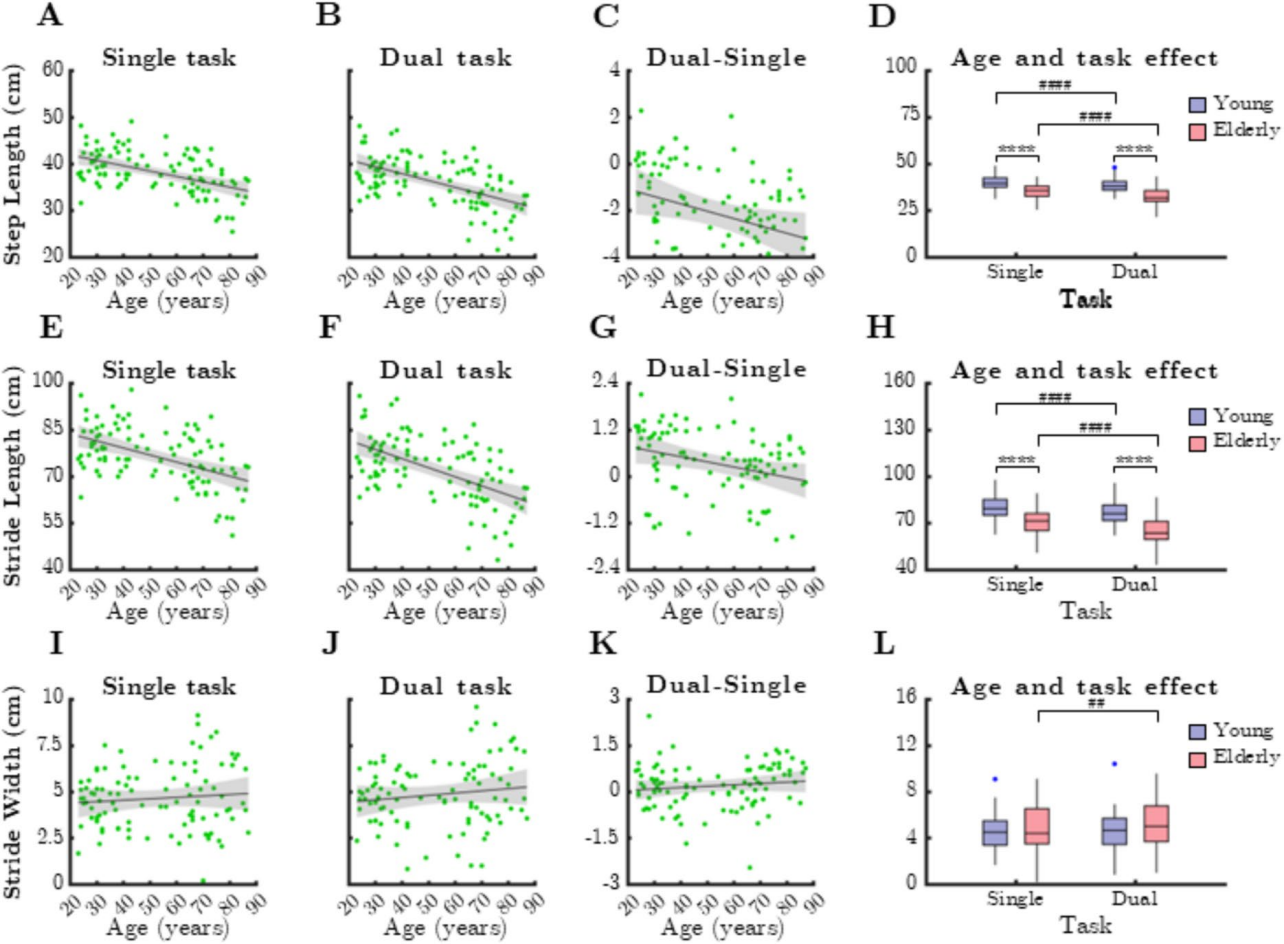
Relationship between age and height normalized spatial parameters of gait during single and dual task. SteLen: step length, StrLen: stride length, StrW: stride width. Black lines and grey shaded areas on the scatterplot represent the trendline and its 95% confidence interval, respectively. Box-whisker plots show data for the young (*n* = 60) and elderly (*n* = 41) groups separately in light purple-blue and light pink-red, respectively. Blue and red dots denote statistical outliers. **A** Mean SteLen corresponding to single task significantly decreases with age (Spearman’s *p* < 0.05). **B** Mean SteLen corresponding to dual task significantly decreases with age (Spearman’s *p* < 0.05). **C** Difference in mean SteLen between single and dual task significantly decreases with age (Spearman’s *p* < 0.05). **D** Impact of age group and task condition on the mean SteLen. **E** Mean StrLen corresponding to single task significantly decreases with age (*p* < 0.05). **F** Mean StrLen corresponding to dual task significantly decreases with age (Spearman’s *p* < 0.05). **G** Difference in mean StrLen between single and dual task significantly decreases with age (Spearman’s *p* < 0.05). **H** Impact of age group and task condition on the median of mean StrLen. **I** Age-related changes in mean StrW, single task. **J** Age-related changes in mean StrW, dual task. **K** Difference in mean StrW between single and dual task significantly decreases with age (Spearman’s *p* < 0.05). **L** Impact of age group and task condition on the median of mean StrW. Panels **D**, **H,** and **L**:; ^****^: *p* < 0.0001, age effect (Mann–Whitney test); ^##^: *p* < 0.01, task effect (paired *t*-test); ^####^: *p* < 0.0001, task effect (Wilcoxon test)

Analysis of spatial gait parameters (Fig. 2) revealed a strong negative correlation between step length and age, observed in both single-task (Fig. 2A, rho =  − 0.487, *p* < 0.0001) and dual-task conditions (Fig. 2B, rho =  − 0.533, *p* < 0.0001). Similar associations were found for stride length (Fig. 2E, rho =  − 0.467, *p* < 0.0001; Fig. 2F, rho =  − 0.524, *p* < 0.0001). Stride width, however, did not correlate with age (*p* > 0.05).

Interestingly, dual-task cost for step length (Fig. 2C, rho =  − 0.319, *p* = 0.0012) and stride length (Fig. 2G, rho =  − 0.324, *p* = 0.0009) also showed significant negative correlations with age, indicating a diminished dual-task effect in older adults. Dual-task cost for stride width exhibited a weak but significant association with age (Fig. 2K, rho = 0.198, *p* = 0.0471).

Comparison between age groups demonstrated that step length and stride length were significantly lower in older adults across all conditions (*p* < 0.0001, Mann–Whitney test). Dual-task conditions also had a pronounced effect on spatial gait parameters (Fig. 2D, H), with significant reductions observed during dual-task walking (*p* < 0.0001, Wilcoxon test). Notably, stride width was significantly lower during dual-task conditions, but only in older adults (Fig. 2L, p = 0.001, paired *t*-test).

### Gait cycle parameters

**Fig. 3:**
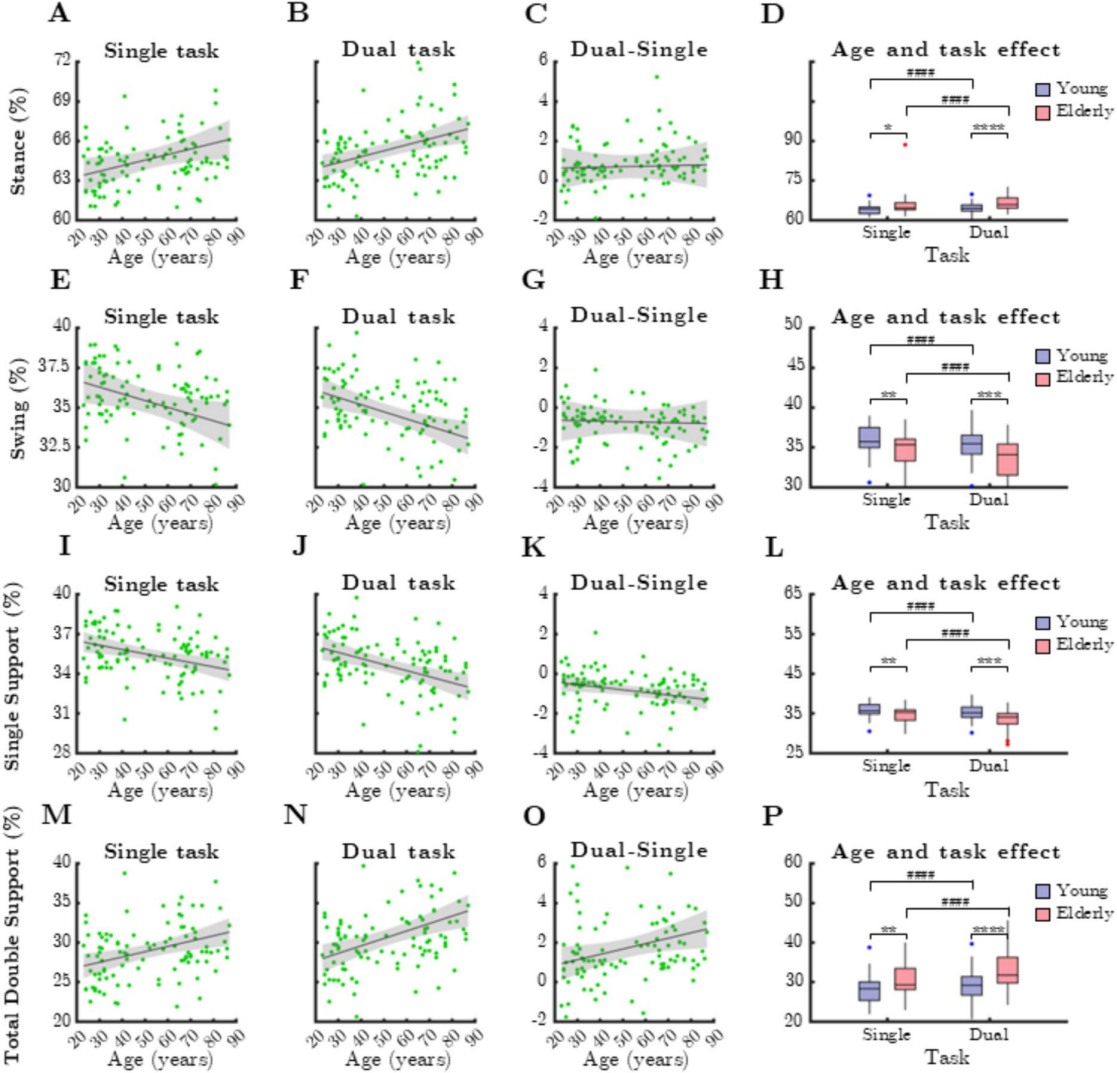
Relationship between age and gait cycle parameters during single and dual task. SSup: single support, TDSup: total double support. Black lines and grey shaded areas on the scatterplot represent the trendline and its 95% confidence interval, respectively. Box-whisker plots show data for the young (*n* = 60) and elderly (*n* = 41) groups separately in light purple-blue and light pink-red, respectively. Blue and red dots denote statistical outliers. **A** Mean stance corresponding to single task significantly increases with age (Spearman’s *p* < 0.05). **B** Mean stance corresponding to dual task significantly increases with age (Spearman’s *p* < 0.05). **C** Difference in mean Stance between single and dual task significantly increases with age (Spearman’s *p* < 0.05). **D** Impact of age group and task condition on the median of stance. **E** Mean swing% corresponding to single task significantly decreases with age (Spearman’s *p* < 0.05). **F** Mean Swing% corresponding to dual task significantly decreases with age (Spearman’s *p* < 0.05). **G** Difference in mean swing between single and dual task significantly decreases with age (Spearman’s *p* < 0.05). **H** Impact of age group and task condition on the median of mean Swing%. **I** Mean SSup corresponding to single task significantly decreases with age (Spearman’s *p* < 0.05). **J** Mean SSup corresponding to dual task significantly decreases with age (Spearman’s *p* < 0.05). **K** Difference in mean SSup between single and dual task significantly decreases with age (Spearman’s *p* < 0.05). **L** Impact of age group and task condition on the median of mean SSup. **M** Mean TDSup corresponding to single task significantly increases with age (Spearman’s *p* < 0.05). **N** Mean TDSup corresponding to dual task significantly increases with age (Spearman’s *p* < 0.05). **(O)** Difference in mean TDSup between single and dual task significantly increases with age (Spearman’s *p* < 0.05). **P** Impact of age group and task condition on the median of mean TDSup. Panels **D**, **H**, **L,** and **P**: ^*^: *p* < 0.05, ^**^: *p* < 0.01, ^***^: *p* < 0.001 and ^****^: *p* < 0.001, age effect (Mann–Whitney test). ^####^: *p* < 0.0001, task effect (Wilcoxon test)

The overall duration of the gait cycle (stride time) was not significantly associated with chronological age. Gait cycle parameters (Fig. 3) demonstrated strong age-related changes. Stance phase duration increased significantly with age during single-task (Fig. 3A, rho = 0.306, *p* = 0.002) and dual-task conditions (Fig. 3B, rho = 0.369, *p* < 0.0001), at the expense of swing phase duration, which decreased accordingly. Dual-task conditions further emphasized these age-related changes (Fig. 3C, G, |*r*|= 0.21, *p* = 0.035).

As single support is a fraction of the swing phase, its age-related changes mirrored those of swing duration (single-task: Fig. 3I, rho =  − 0.321, *p* = 0.001; dual-task: Fig. 3J, rho =  − 0.39, *p* < 0.0001). Conversely, total double support duration increased with age, similar to stance phase (single-task: Fig. 3M, rho = 0.323, *p* = 0.001; dual-task: Fig. 3N, rho = 0.398, *p* < 0.0001).

The dual-single task difference significantly correlated with age for both single support (Fig. 3K, rho =  − 0.286, *p* = 0.004) and total double support (Fig. 3O, rho = 0.309, *p* = 0.0017).

Comparisons between age groups revealed significant differences in stance and swing phases (Fig. 3D, H, single-task: *p* = 0.005, dual-task: *p* < 0.0001, Mann–Whitney test) and single and total double support (Fig. 3L, P, single-task: *p* = 0.003, dual-task: *p* < 0.0001, Mann–Whitney test). Dual-task conditions had a significant effect on all gait cycle parameters in both age groups (*p* < 0.0001, Wilcoxon test).

### Balance control parameters

**Fig. 4:**
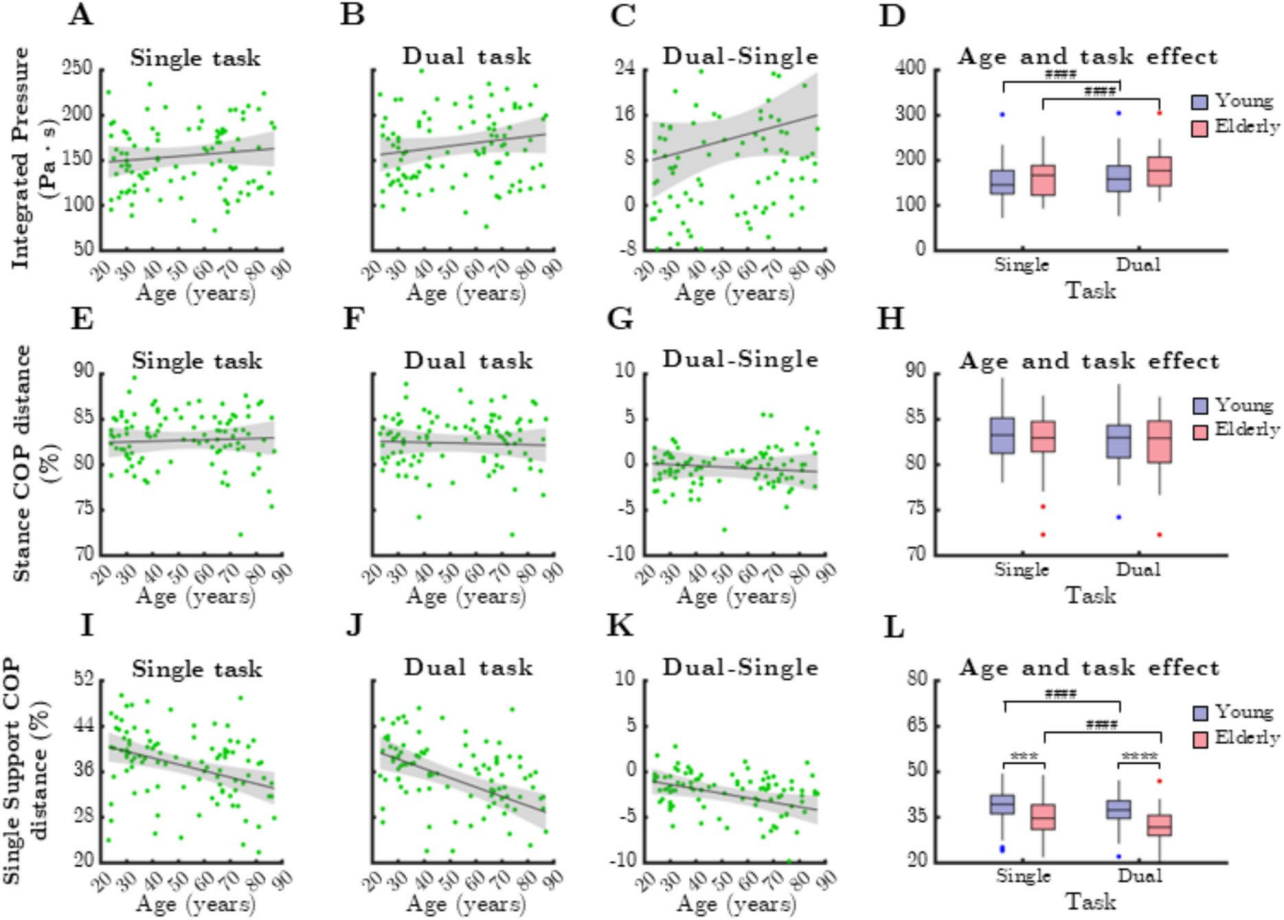
Relationship between age and parameters reflecting control of balance during single and dual task. IntP: integrated pressure, StCOPd: stance center of pressure distance, SSCOPd: single support center of pressure distance. Black lines and grey shaded areas on the scatterplot represent the trendline and its 95% confidence interval, respectively. Boxwhisker plots show data for the young (*n *= 60) and elderly (*n* = 41) groups separately in light purple-blue and light pink-red, respectively. Blue and red dots denote statistical outliers. (**A**) Age-related changes in mean IntP, single task. (**B**) Agerelated changes in mean IntP, dual task. (**C**) Difference in mean IntP between single and dual task, as a function of age. (**D**) Impact of age group and task condition on the median of mean IntP. (**E**) Age-related changes in mean StCOPd, single task. (**F**) Age-related changes in mean StCOPd, dual task. (**G**) Difference in mean StCOPd between single and dual task as a function of age. (**H**) Impact of age group and task condition on the median of mean StCOPd. (**I**) Mean SSCOPd corresponding to dual task significantly decreases with age (Spearman’s *p *<0.05). (**J**) Mean SSCOPd corresponding to dual task significantly decreases with age (Spearman’s *p *<0.05). (**K**) Difference in mean SSCOPd between single and dual task significantly decreases with age (Spearman’s *p* <0.05). Black line and grey shaded area represents the trendline and its 95% confidence interval, respectively. (**L**) Impact of age group and task condition on the median of mean SSCOPd. Panels D, H and L:; *** : *p *<0.001 and **** : *p *<0.0001, age effect (Mann-Whitney test); ##: *p *<0.01, task effect (Wilcoxon-test); ####: *p *<0.0001, task effect (Wilcoxon test)

Figure 4 illustrates the relationship between mean balance control parameters and age under single- and dual-task conditions. Mean integrated pressure showed a slight increase with age (Fig. 4 A), which was further emphasized by dual-task walking (Fig. 4B, C). However, age groups did not differ significantly (*p* > 0.05). The difference between walking conditions was significant (*p* < 0.0001, Wilcoxon test), indicating a task-related impact on balance control.

Stance COP distance remained stable across age groups and task conditions (Fig. 4E, F, G). However, single support COP distance showed a significant negative correlation with age (Fig. 4I, rho =  − 0.381, *p* < 0.0001; Fig. 4 J, rho =  − 0.473, *p* < 0.0001), with older adults demonstrating shorter COP distances. Additionally, the dual-task effect on single support COP distance diminished with age (Fig. 4 K, rho =  − 0.322, *p* = 0.001).

Comparing age groups, single support COP distance was significantly lower in older adults during both single-task (*p* = 0.001) and dual-task conditions (*p* < 0.0001, Mann–Whitney test) (Fig. 4L). Furthermore, dual-task conditions significantly reduced single support COP distance in both age groups (*p* < 0.0001, Wilcoxon test), highlighting the impact of cognitive load on balance control.

### Left–right ratio index of gait asymmetry

One of the parameters used to characterize gait asymmetry was the RI, which quantifies differences in gait variables between the left and right legs, as defined in Eq. 1. The association between RI and age, as well as differences between age groups, was evaluated for multiple gait parameters. Descriptive statistics for these measures are presented in Table 4.

### Temporal gait parameters

**Fig. 5:**
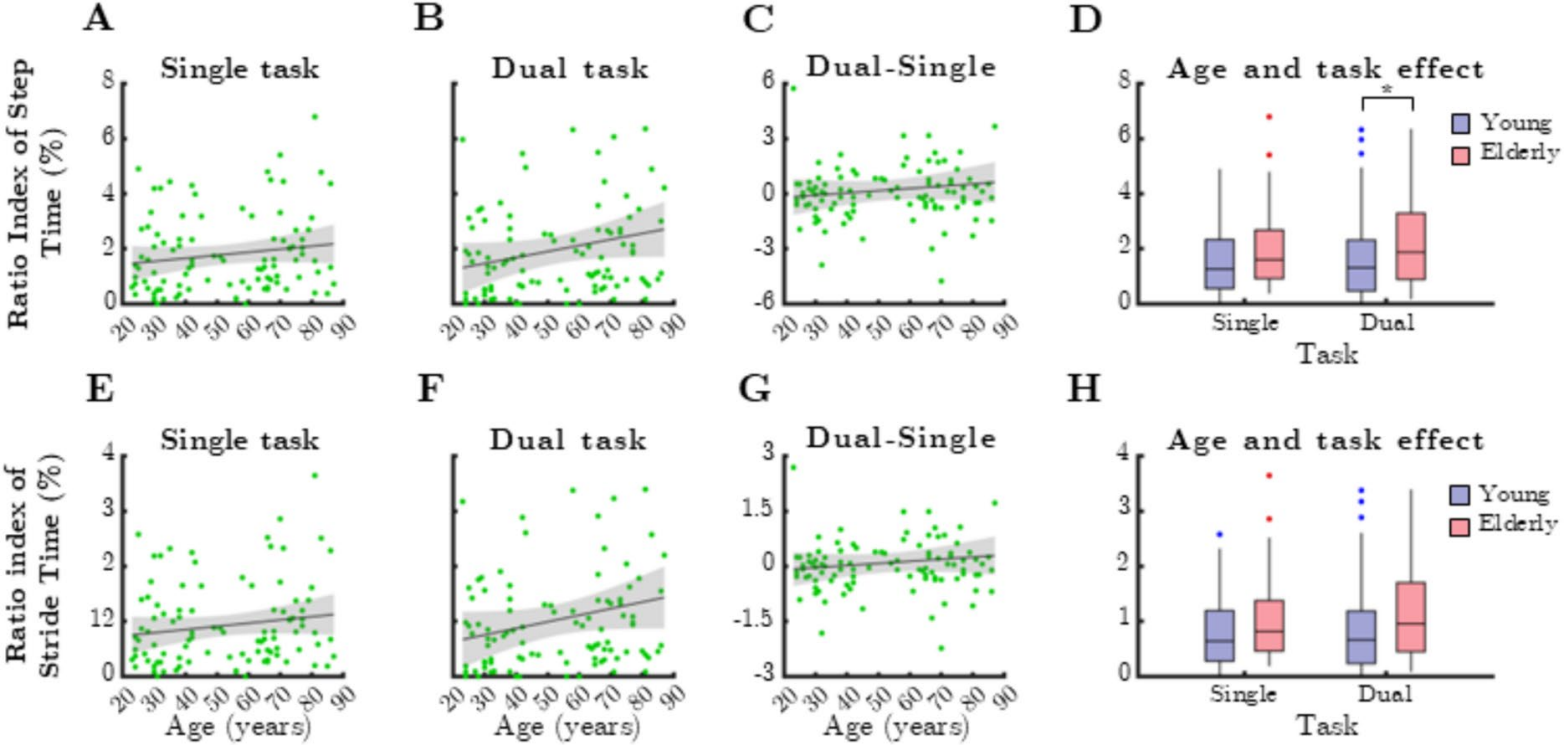
Relationship between age and mean ratio index (RI) of temporal gait parameters during single and dual task. SteTim: step time, StrTim: stride time. Black lines and grey shaded areas on the scatterplot represent the trendline and its 95% confidence interval, respectively. Box-whisker plots show data for the young (*n* = 60) and elderly (*n* = 41) groups separately in light purple-blue and light pink-red, respectively. Blue and red dots denote statistical outliers. **A** Age-related changes in mean RI(SteTim), single task. **B** Mean RI(SteTim) corresponding to dual task significantly increases with age (Spearman’s *p* < 0.05). **C** Difference in mean RI(SteTim) between single and dual task, as a function of age. **D** Impact of age group and task condition on the median of mean RI(SteTim). **E** Age-related changes in mean RI(StrTim), single task. **F** Mean RI(StrTim) corresponding to dual task significantly increases with age (Spearman’s *p* < 0.05). **G** Difference in mean RI(StrTim) between single and dual task, as a function of age. **H** Impact of age group and task condition on the median of mean RI(StrTim). Panel **D**: ^*^p < 0.05: age effect (Mann–Whitney test)

The ratio index of step time exhibited a significant age-related increase under dual-task conditions (Fig. 5B, Spearman’s rho =  − 0.4476, *p* < 0.0001) and was significantly higher among older adults during this condition (Fig. 5D, p = 0.028, Mann–Whitney test). However, dual-task cost did not correlate with chronological age (*p* > 0.05). Similar trends were observed for the ratio index of stride time (Fig. 5E–H), though no significant associations were found between age, task condition, or group differences for this parameter.

### Spatial gait parameters

**Fig. 6:**
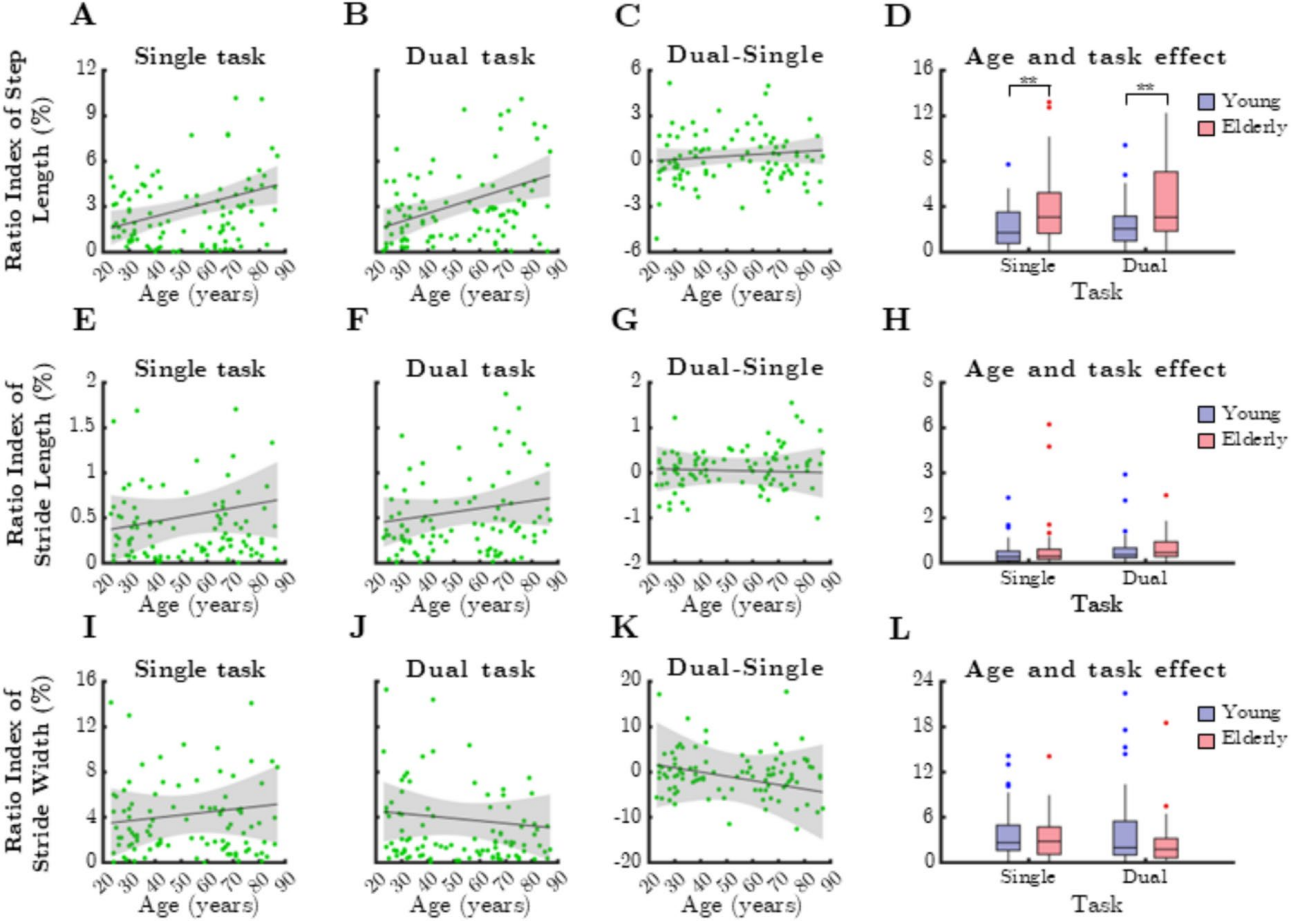
Relationship between age and mean ratio index (RI) of spatial gait parameters during single and dual task. SteLen: step length, StrLen: stride length, StrW: stride width. Black lines and grey shaded areas on the scatterplot represent the trendline and its 95% confidence interval, respectively. Box-whisker plots show data for the young (*n* = 60) and elderly (*n* = 41) groups separately in light purple-blue and light pink-red, respectively. Blue and red dots denote statistical outliers. **A** Mean RI(SteLen) corresponding to dual task significantly increases with age (Spearman’s *p* < 0.05). **B** Mean RI(SteLen) corresponding to dual task significantly increases with age (Spearman’s *p* < 0.05). **C** Difference in mean RI(SteLen) between single and dual task, as a function of age. **D** Impact of age group and task condition on the median of mean RI(SteLen). **E** Age-related changes in mean RI(StrLen), single task. **F** Age-related changes in mean RI(StrLen), dual task. **G** Difference in mean RI(StrLen) between single and dual task, as a function of age. **H** Impact of age group and task condition on the median of mean RI(SteLen). **I** Age-related changes in mean StrW, single task. **J** Age-related changes in mean StrW, dual task. **K** Difference in mean StrW between single and dual task, as a function of age. **L** Impact of age group and task condition on the median of mean StrW. Panel **D**: ^**^: *p* < 0.01, age effect (Mann–Whitney test)

Analysis of RI derived from spatial gait parameters (Fig. 6) showed a positive correlation between RI of step length and chronological age for both task conditions (Fig. 6A: single task, rho = 0.268, *p* = 0.007; Fig. 6B: dual task, rho = 0.324, *p* = 0.001). However, RI of stride length and RI of stride width did not exhibit significant age-related associations. Similarly, dual-task cost of RI values for spatial gait parameters (step length, stride length, and stride width) did not correlate with age (Fig. 6C, G, and K).

When comparing age groups, RI of step length was significantly greater in older adults (Fig. 6D, single task: *p* = 0.003; dual task: *p* = 0.006, Mann–Whitney test). In contrast, stride length and stride width did not differ significantly between age groups. Furthermore, RI of spatial gait parameters did not show significant differences between single- and dual-task conditions in either age group.

### Gait cycle parameters

**Fig. 7:**
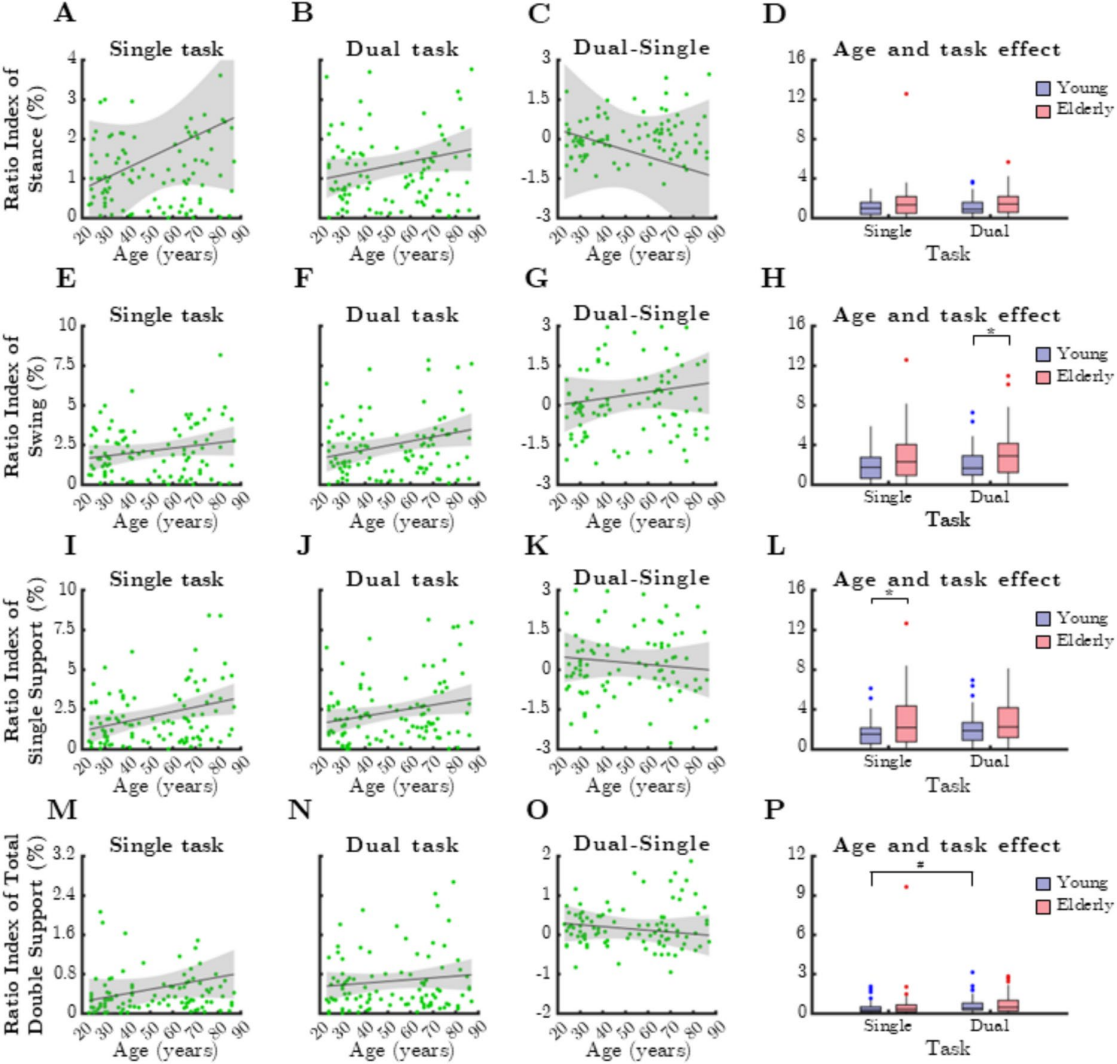
Relationship between age and mean ratio index (RI) of gait cycle parameters during single and dual task. SSup: single support, TDSup: total double support. Black lines and grey shaded areas on the scatterplot represent the trendline and its 95% confidence interval, respectively. Box-whisker plots show data for the young (*n* = 60) and elderly (*n* = 41) groups separately in light purple-blue and light pink-red, respectively. Blue and red dots denote statistical outliers. **A** Age-related changes in mean RI(Stance), single task. **B** Mean RI(Stance) corresponding to dual task significantly increases with age (Spearman’s *p* < 0.05). **C** Difference in mean RI(Stance) between single and dual task, as a function of age. **D** Impact of age group and task condition on the median of mean RI(Stance). **E** Age-related changes in mean RI(Swing), single task. **F** Mean RI(Swing) corresponding to dual task significantly increases with age (Spearman’s *p* < 0.05). **G** Difference in mean RI(Swing) between single and dual task, as a function of age. **H** Impact of age group and task condition on the median of mean RI(Swing). **I** Mean RI(SSup) corresponding to single task significantly increases with age (Spearman’s *p* < 0.05). **J** Age-related changes in mean RI(SSup), dual task. **K** Difference in mean RI(SSup) between single and dual task, as a function of age. **L** Impact of age group and task condition on the median of mean RI(SSup). **M** Age-related changes in mean RI(TDSup), single task. **N** Age-related changes in mean RI(TDSup), dual task. **O** Difference in mean RI(TDSup) between single and dual task, as a function of age. **P** Impact of age group and task condition on the median of mean RI(TDSup). Panels **H** and **L**: ^*^: *p* < 0.05, age effect (Mann–Whitney test). ^#^: *p* < 0.05, task effect (Wilcoxon test)

Ratio indices of gait cycle parameters exhibited a general tendency to increase with age, but substantial individual variability weakened these correlations (Fig. 7). The RI of swing phase increased significantly with age during dual-task conditions (Fig. 7B, rho = 0.197, *p* = 0.048), similar to swing phase, though these associations were not significant for stance phase and under single-task conditions. The dual-task effect did not further emphasize the age-related increase in asymmetry (Fig. 7C and G).

For single support in the single-task condition, RI increased with age (Fig. 7I, rho = 0.246, *p* = 0.013) and was significantly higher in older adults (Fig. 7L, p = 0.016, Mann–Whitney test). In contrast, double support only showed a significant task-related effect in the younger group (Fig. 7P, p = 0.003, Wilcoxon test). No significant age-related associations were observed in the dual-task cost of gait cycle parameters.

### Balance control parameters

**Fig. 8:**
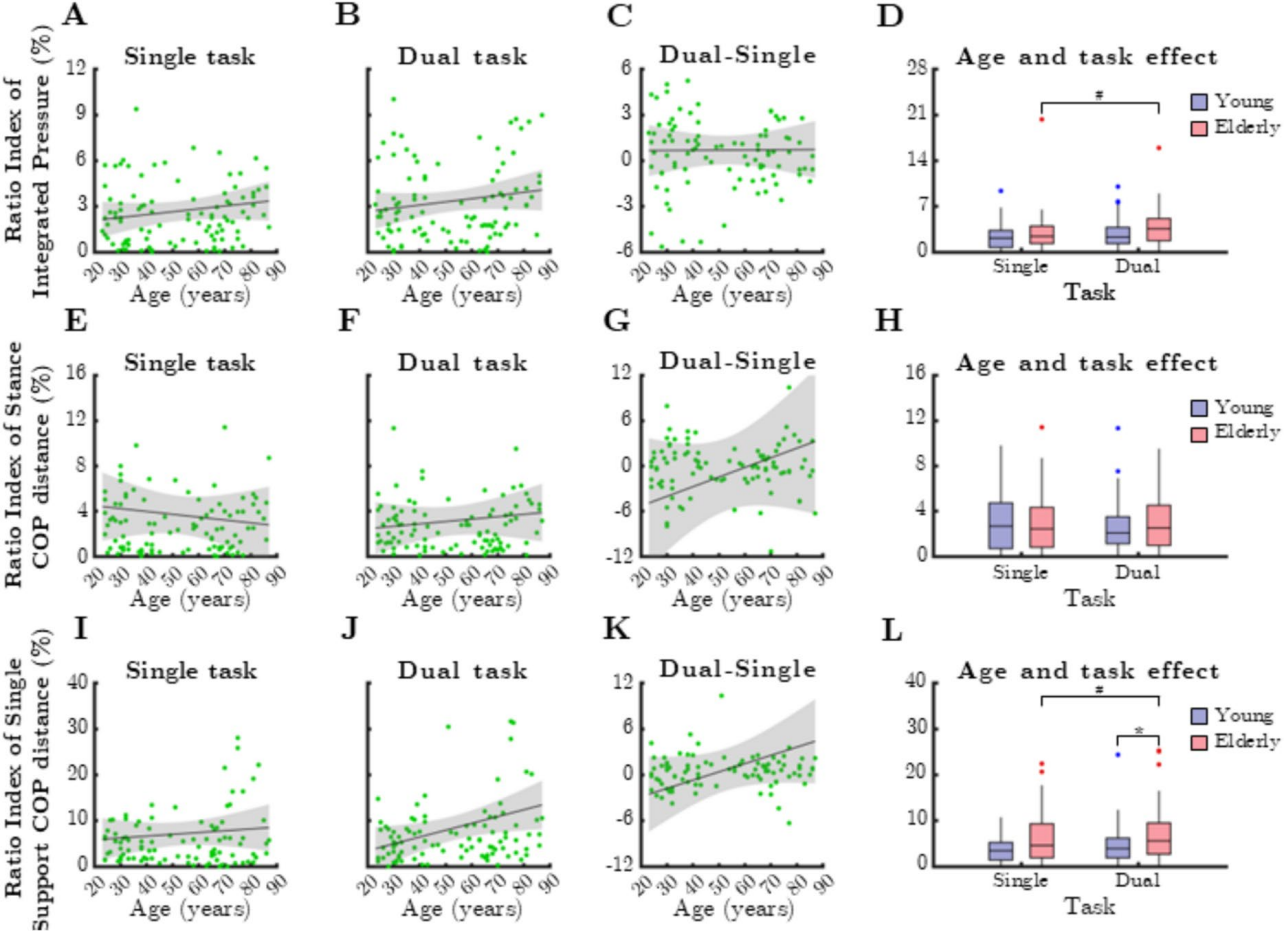
Relationship between age and mean ratio index (RI) parameters reflecting control of balance during single and dual task. IntP: integrated pressure, StCOPd: stance center of pressure distance, SSCOPd: single support center of pressure distance. Black lines and grey shaded areas on the scatterplot represent the trendline and its 95% confidence interval, respectively. Box-whisker plots show data for the young (*n* = 60) and elderly (*n* = 41) groups separately in light purple-blue and light pink-red, respectively. Blue and red dots denote statistical outliers. **A** Age-related changes in mean RI(IntP), single task. **B** Age-related changes in mean RI(IntP), dual task. **C** Difference in mean RI(IntP) between single and dual task, as a function of age. **D** Impact of age group and task condition on the median of mean RI(IntP). **E** Age-related changes in mean RI(StCOPd), single task. **F** Age-related changes in mean RI(StCOPd), dual task. **G** Difference in mean RI(StCOPd) between single and dual task, as a function of age. **H** Impact of age group and task condition on the median of mean RI(StCOPd). **I** Age-related changes in mean RI(SSCOPd), single task. **J** Mean RI(SSCOPd) corresponding to dual task significantly increases with age (Spearman’s *p* < 0.05). **K** Difference in mean RI(SSCOPd) between single and dual task, as a function of age. **L** Impact of age group and task condition on the median of mean RI(SSCOPd). Panels **D** and **L**: ^*^: *p* < 0.05, age effect (Mann–Whitney test). ^#^: *p* < 0.05, task effect (Wilcoxon test)

The relationship between mean RI of balance control parameters and age during single- and dual-task conditions is shown in Fig. 8. RI values calculated for mean integrated pressure (Fig. 8A), stance COP distance (Fig. 8E), and single support COP distance (Fig. 8I) did not vary with age. In case of dual task, higher single support COP distance values were measured for older participants (Fig. 8J, rho = 0.256, *p* = 0.010). However, dual-task cost varied independently from age (Fig. 8C, G, K).

A significant age group effect was observed for mean RI of stance COP distance, but only during dual-task conditions (Fig. 8L, p = 0.040, Mann–Whitney test). Additionally, task effects were significant in older adults for mean RI of integrated pressure (Fig. 8D, p = 0.029, Wilcoxon test) and single support COP distance (Fig. 8L, p = 0.023).

### Symmetry index of gait pattern

To provide a comprehensive characterization of gait asymmetry, a Symmetry Index (SI) was calculated for each walking session based on Eq. 2, which normalizes the absolute deviation between the left and right legs to their sum. The association between SI and age, as well as differences between age groups, was analyzed using the same approach applied for the Ratio Index (RI). Descriptive statistics for SI values across gait parameters are presented in Table 4.

### Temporal gait parameters

**Fig. 9:**
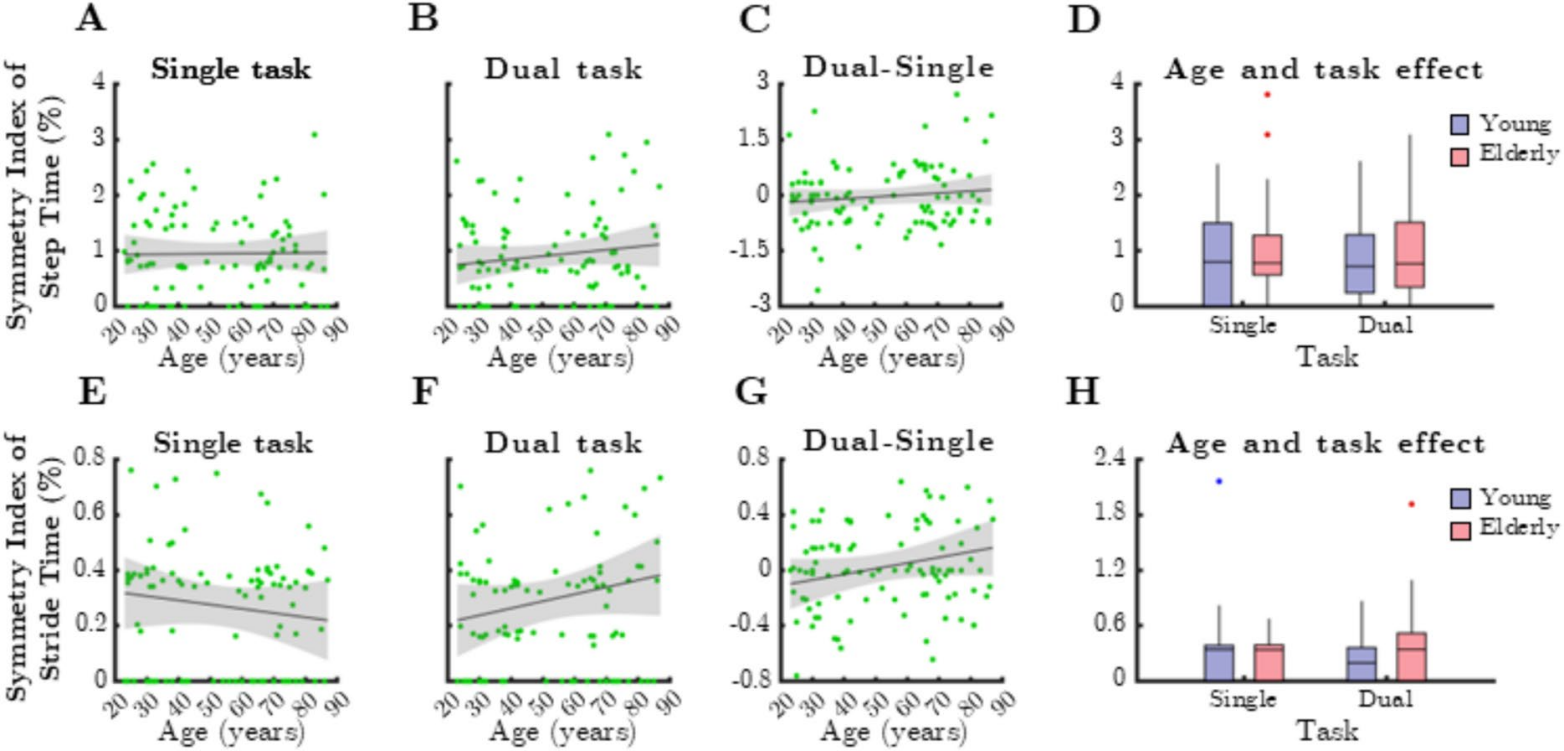
Relationship between age and median symmetry index (SI) of temporal gait parameters during single and dual task. SteTim: mean step time, StrTim: mean stride time. Black lines and grey shaded areas on the scatterplot represent the trendline and its 95% confidence interval, respectively. Box-whisker plots show data for the young (*n* = 60) and elderly (*n* = 41) groups separately in light purple-blue and light pink-red, respectively. Blue and red dots denote statistical outliers. **A** Age-related changes in median SI(SteTim), single task. **B** Age-related changes in median SI(SteTim), dual task. **C** Difference in median SI(SteTim) between single and dual task, as a function of age. **D** Impact of age group and task condition on the median of median SI(SteTim). **E** Age-related changes in median SI(StrTim), single task. **F** Age-related changes in median SI(StrTim), dual task. **G** Difference in median SI(StrTim) between single and dual task, as a function of age. **H** Impact of age group and task condition on the median of median SI(StrTim)

None of the temporal gait parameters demonstrated greater asymmetry in older adults in terms of median SI (Fig. 9). There was no significant correlation between age and step time asymmetry in either single-task (Fig. 9A, B) or dual-task conditions (Fig. 9C). Similarly, stride time asymmetry did not exhibit a significant relationship with age (Fig. 9E–G) or group differences, indicating that temporal gait asymmetry remains stable across aging.

### Spatial gait parameters

**Fig. 10:**
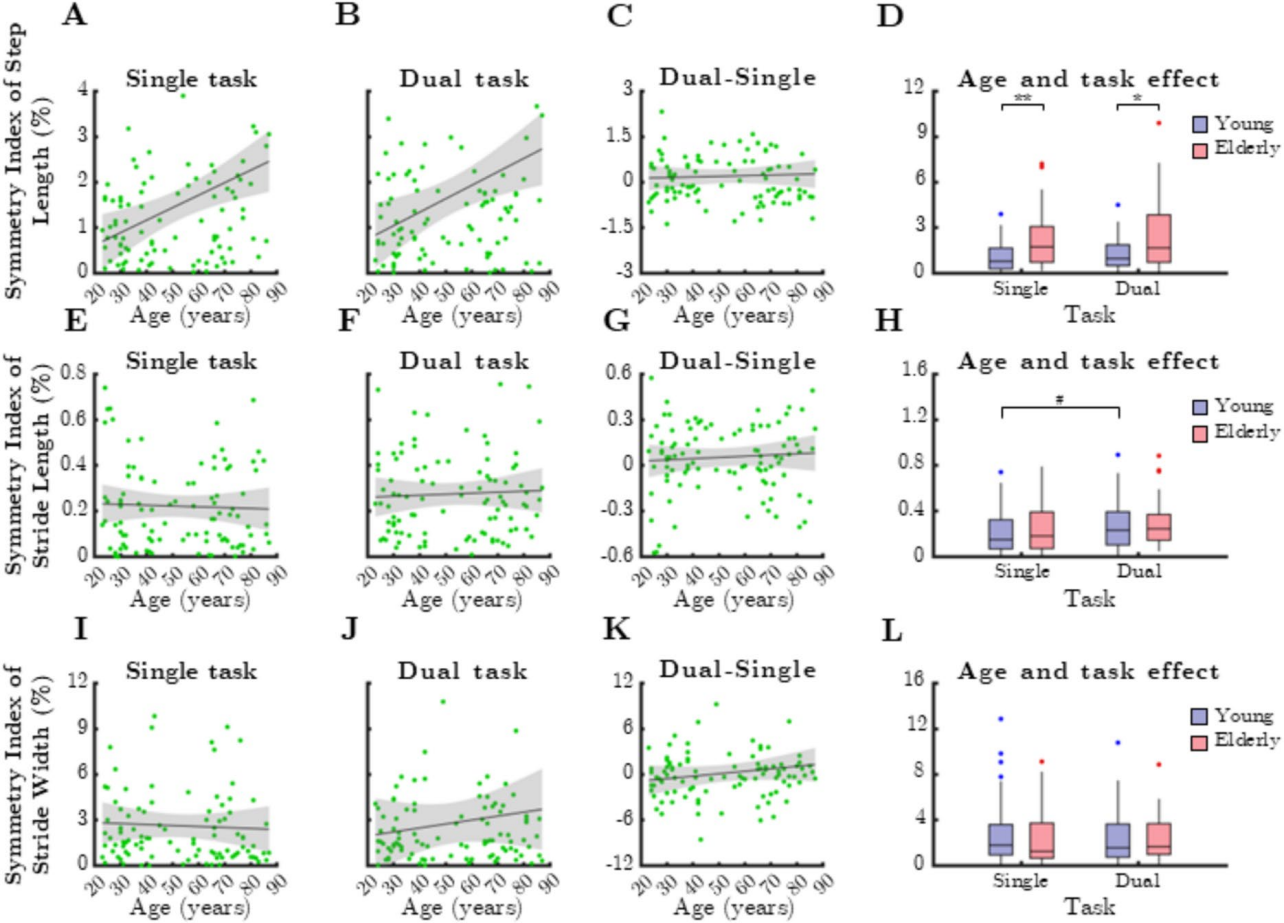
Relationship between age and median symmetry index (SI) of height normalized spatial gait parameters during single and dual task. SteLen: step length, StrLen: stride length, StrW: stride width. Black lines and grey shaded areas on the scatterplot represent the trendline and its 95% confidence interval, respectively. Box-whisker plots show data for the young (*n* = 60) and elderly (*n* = 41) groups separately in light purple-blue and light pink-red, respectively. Blue and red dots denote statistical outliers. **A** Median SI(SteLen) corresponding to single task significantly increases with age (Spearman’s *p* < 0.05). **B** Median SI(SteLen) corresponding to dual task significantly increases with age (Spearman’s *p* < 0.05). **C** Difference in median SI(SteLen) between single and dual task, as a function of age. **D** Impact of age group and task condition on the median of median SI(SteLen). **E** Age-related changes in mean SI(StrLen), single task. **F** Age-related changes in mean SI(StrLen), dual task. **G** Difference in mean SI(StrLen) between single and dual task, as a function of age. **H** Impact of age group and task condition on the median of median SI(SteLen). **I** Age-related changes in median SI(StrW), single task. **J** Age-related changes in median SI(StrW), dual task. **K** Difference in median SI(StrW) between single and dual task, as a function of age. **L** Impact of age group and task condition on the median of median SI(StrW). Panels **D** and **H**: ^*^p < 0.05: age effect (Mann–Whitney test); ^**^: *p* < 0.01: age effect (Mann–Whitney test); ^#^: *p* < 0.05 (Wilcoxon test)

Analysis of median SI for spatial gait parameters (Fig. 10) revealed a strong positive correlation between step length asymmetry and chronological age, observed in both single-task (Fig. 10A, rho = 0.328, *p* = 0.0008) and dual-task conditions (Fig. 10B, rho = 0.284, *p* = 0.0041). However, stride length (Fig. 10E, F) and stride width (Fig. 10I, J) asymmetries did not show a significant correlation with age (*p* > 0.05).

When comparing age groups, step length asymmetry was significantly greater in older adults for both single-task (*p* = 0.0012, Mann–Whitney test) and dual-task conditions (*p* = 0.0184) (Fig. 10D). While gait asymmetry tended to increase during dual-task walking, this effect was only statistically significant for stride length asymmetry in the younger group (Fig. 10H, p = 0.0267, Wilcoxon test). Stride width asymmetry did not show significant age- or task-related differences (Fig. 10I–L). Additionally, dual-task cost did not correlate with age for spatial gait parameters.

### Gait cycle parameters

**Fig. 11:**
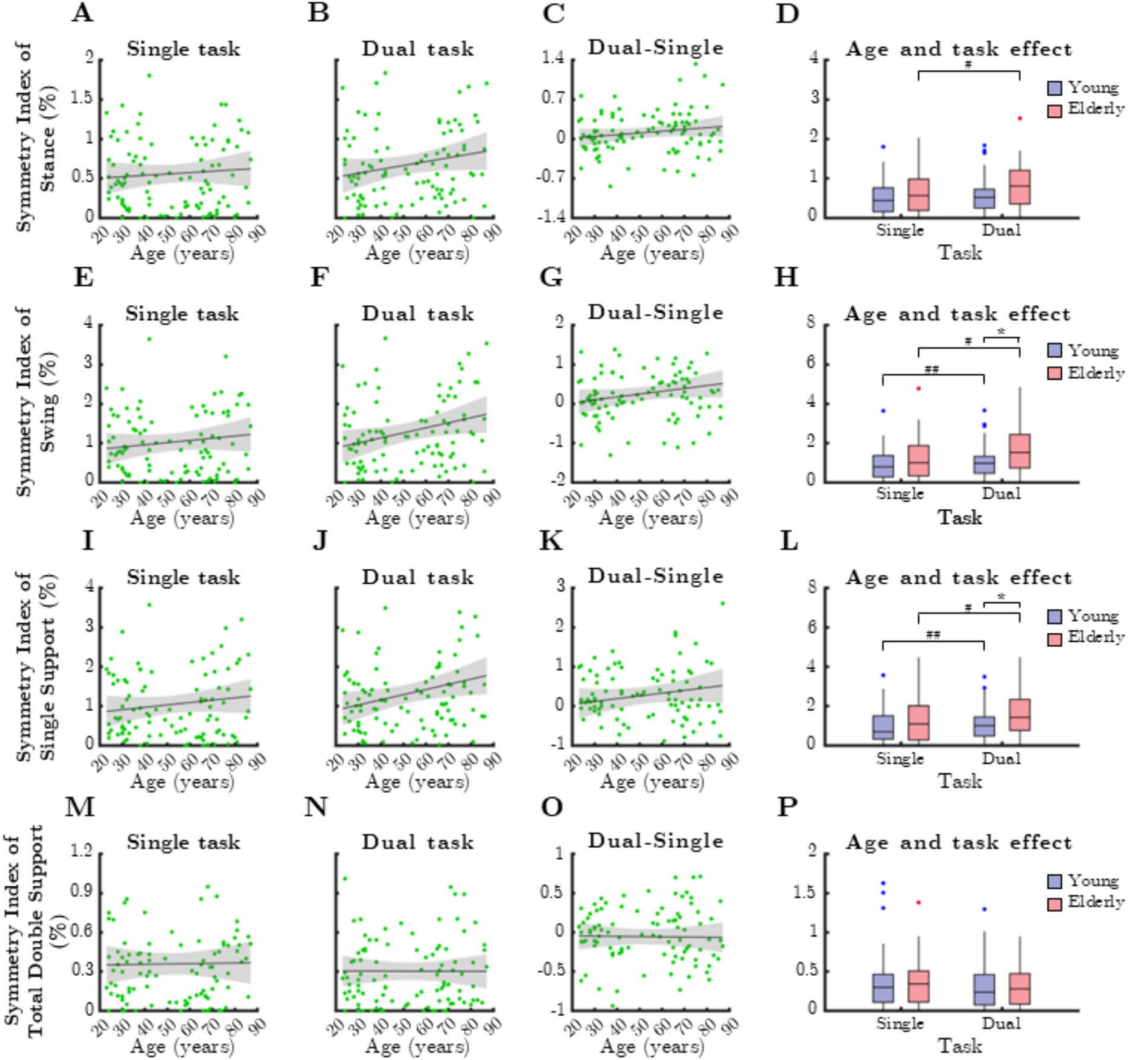
Relationship between age and median symmetry index (SI) of gait cycle parameters during single and dual task. SSup: single support, TDSup: total double support. Black lines and grey shaded areas on the scatterplot represent the trendline and its 95% confidence interval, respectively. Box-whisker plots show data for the young (*n* = 60) and elderly (*n* = 41) groups separately in light purple-blue and light pink-red, respectively. Blue and red dots denote statistical outliers. **A** Age-related changes in median SI(Stance), single task. **B** Age-related changes in median SI(Stance), dual task. **C** Difference in median SI(Stance) between single and dual task, as a function of age. **D** Impact of age group and task condition on the median of median SI(Stance). **E** Age-related changes in median SI(Swing), single task. **F** Median SI(Swing) corresponding to dual task significantly increases with age (Spearman’s *p* < 0.05). **G** Difference in median SI(Swing) between single and dual task, as a function of age. **H** Impact of age and task condition on the median of median SI(Swing). **I** Age-related changes in median SI(SSup), dual task. **J** Age-related changes in median SI(SSup), dual task. **K** Difference in median SI(SSup) between single and dual task, as a function of age. **L** Impact of age group and task condition on the median of median SI(SSup). **M** Age-related changes in median SI(TDSup), single task. **N** Age-related changes in median SI(TDSup), dual task. **O** Difference in median SI(TDSup) between single and dual task, as a function of age. **P** Impact of age group and task condition on the median of median SI(TDSup). Panels **D** and **H**: ^*^p < 0.05: age effect (Mann–Whitney test); ^#^: *p* < 0.01, task effect (Wilcoxon-test); ^##^: *p* < 0.01, task effect (Wilcoxon test)

The median SI of gait cycle parameters is presented in Fig. 11. Swing phase asymmetry exhibited a weak but significant correlation with age in dual-task conditions (Fig. 11F, rho = 0.239, *p* = 0.0161), whereas no other gait cycle parameters were significantly associated with age.

When comparing age groups, older adults demonstrated greater asymmetry for swing phase (Fig. 11H, p = 0.0064, Mann–Whitney test) and single support phase (Fig. 11L, p = 0.0138), whereas other gait cycle parameters remained unaffected. Cognitive dual-tasking increased asymmetry in several gait cycle parameters, with significant effects observed in: (1) Stance phase asymmetry in older adults (Fig. 11D, p = 0.0193, Wilcoxon test); (2) Swing phase asymmetry in both younger (Fig. 11H, p = 0.023) and older adults (*p* = 0.0048); and (3) Single support asymmetry in both younger (Fig. 11L, p = 0.0303) and older adults (*p* = 0.0438).

### Balance control parameters

**Fig. 12:**
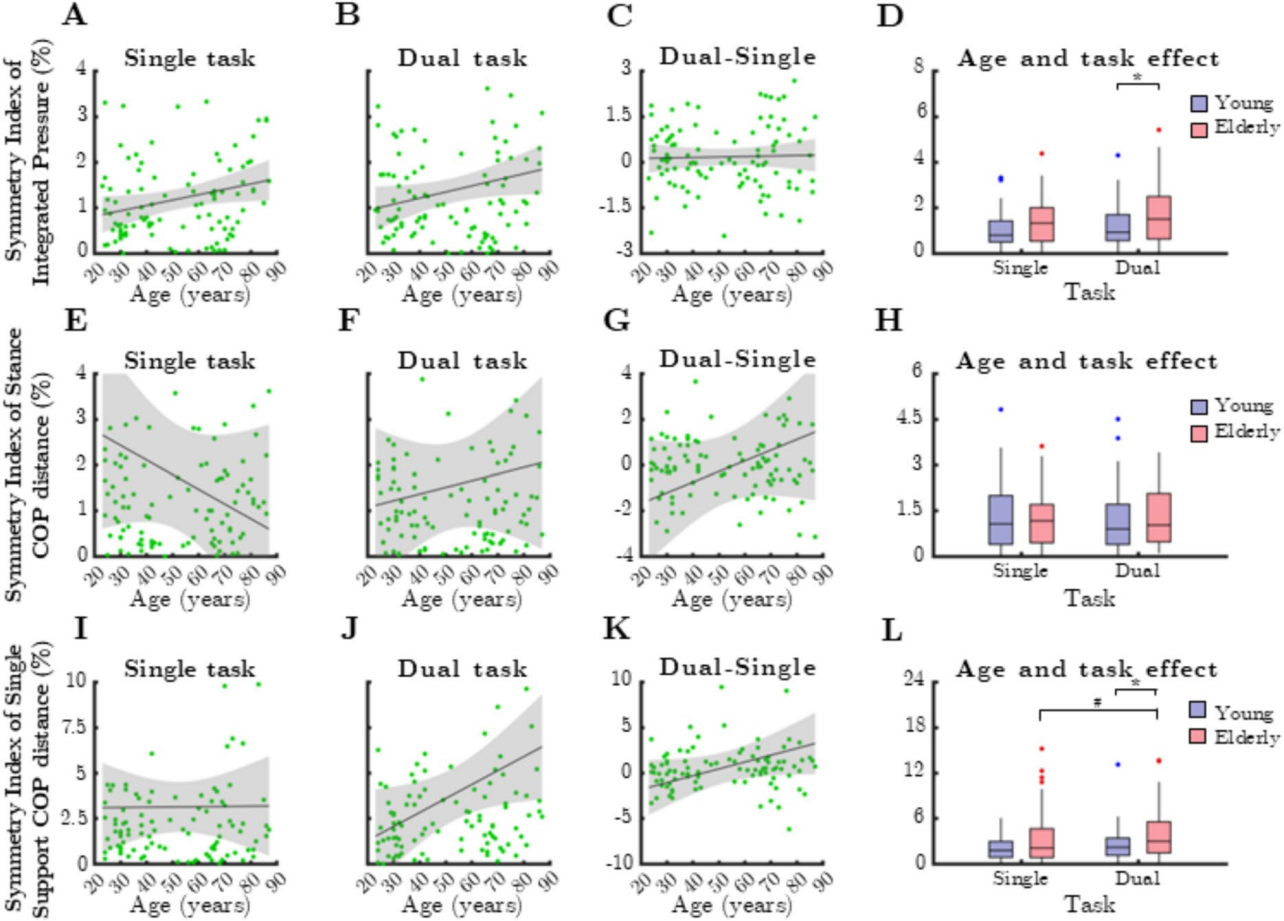
Relationship between age and median symmetry index (SI) parameters reflecting control of balance during single and dual task. IntP: integrated pressure, StCOPd: stance center of pressure distance, SSCOPd: single support center of pressure distance. Black lines and grey shaded areas on the scatterplot represent the trendline and its 95% confidence interval, respectively. Box-whisker plots show data for the young (*n* = 60) and elderly (*n* = 41) groups separately in light purple-blue and light pink-red, respectively. Blue and red dots denote statistical outliers. **A** Age-related changes in median SI(IntP), single task. **B** Median SI(IntP) corresponding to dual task significantly increases with age (Spearman’s *p* < 0.05). **C** Difference in median SI(IntP) between single and dual task, as a function of age. **D** Impact of age group and task condition on the median of median SI(IntP). **E** Age-related changes in median SI(StCOPd), single task. **F** Age-related changes in median SI(StCOPd), dual task. **G** Difference in median SI(StCOPd) between single and dual task, as a function of age. **H** Impact of age group and task condition on the median of median SI(StCOPd). **I** Age-related changes in median SI(SSCOPd), single task. **J** Median SI(SSCOPd) corresponding to dual task significantly increases with age (Spearman’s *p* < 0.05). **K** Difference in median SI(SSCOPd) between single and dual task significantly increases with age (Spearman’s *p* < 0.05). **L** Impact of age group and task condition on the median of median SI(SSCOPd), Panels **D** and **L**: ^*^: *p* < 0.05, age effect (Mann–Whitney test). ^#^: *p* < 0.05, task effect (Wilcoxon test)

Figure 12 illustrates the relationship between median SI of balance control parameters and age under both single-task and dual-task conditions. Mean integrated pressure asymmetry significantly increased with age, but only in the single-task condition (Fig. 12A, rho = 0.252, *p* = 0.011). However, older adults demonstrated greater asymmetry in the dual-task condition (Fig. 12D, p = 0.0382, Mann–Whitney test).

No significant age-related or group effects were found for stance COP distance asymmetry (Fig. 12E–H). In contrast, single support COP distance asymmetry exhibited a strong age-related correlation during dual-task conditions (Fig. 12J, rho = 0.2824, *p* = 0.0042), whereas no such relationship was observed in single-task walking. Dual-task cost for this parameter also showed a statistically significant positive association with age (Fig. 12K, rho = 0.2015, *p* = 0.0434).

Comparing age groups, single support COP distance asymmetry was significantly greater in older adults during dual-task conditions (Fig. 12L, p = 0.0467). Furthermore, within the older adult group, asymmetry was significantly higher in dual-task than in single-task conditions (*p* = 0.0315, Wilcoxon test), suggesting that cognitive load disproportionately affects balance asymmetry in aging individuals.

## Discussion

This study provides a comprehensive analysis of age-related and cognitive load-induced gait alterations, with a particular focus on gait asymmetry, using Ratio Index (RI) and Symmetry Index (SI) metrics. Our findings demonstrate that gait asymmetry increases with age, particularly in spatial and balance-related parameters, and that cognitive load exacerbates these asymmetries. These results underscore the importance of integrating dual-task gait assessments into aging research and highlight gait asymmetry as a potential biomarker of functional and cognitive decline.

### Implications for the Semmelweis Study

This pilot investigation serves as an essential step in optimizing the gait assessment protocol for large-scale longitudinal analyses within the Semmelweis Study framework, ensuring the integration of objective mobility assessments into the study’s broader efforts to investigate aging-related functional decline and cognitive health trajectories. The findings provide valuable insights into age-related gait asymmetries and confirm the feasibility of using high-throughput gait analysis tools to detect early mobility and cognitive decline. Given the Semmelweis Study’s focus on healthy brain aging [51], the implementation of dual-task gait analysis presents a unique opportunity to identify individuals experiencing accelerated aging and those at increased risk for functional and cognitive impairments at an early stage. By refining the methodological framework for gait assessments, this study lays the foundation for integrating spatiotemporal gait parameters, asymmetry indices, and cognitive-motor interactions into routine functional evaluations within the Semmelweis Study. The ability to quantify gait asymmetry across a large, diverse workplace cohort will enable the investigation of longitudinal changes in mobility [52] and their relationship to vascular health, metabolic risk factors, and cognitive function—all of which are central priorities of the Semmelweis Study’s aging research agenda. Furthermore, this study highlights step length asymmetry as a potential marker of aging-related motor decline, suggesting that gait-based screening tools could be developed to identify subclinical neurovascular and musculoskeletal impairments. These findings align with the broader mission of the Semmelweis Study to develop early intervention strategies for preserving functional independence and preventing age-related disability in the workforce. Given the study’s emphasis on workplace health promotion, incorporating gait analysis into periodic health assessments for Semmelweis University employees could offer a novel approach to tracking mobility and cognitive health over time. The identification of gait-related biomarkers could also support personalized preventive measures, such as tailored exercise programs, workplace modifications, or cognitive-motor training interventions, aimed at reducing fall risk and enhancing neuromotor resilience in aging employees.

### Age-related changes in gait performance

Consistent with previous studies, we observed a significant decline in gait speed, step length, and stride length with age, with older adults walking significantly slower than younger adults [53–56]. This decline was particularly pronounced under dual-task conditions, supporting the idea that aging is associated with reduced gait adaptability and diminished cognitive-motor integration. The strong negative correlation between age and gait speed, one of the most widely recognized indicators of functional mobility, reinforces its role as a key predictor of fall risk, frailty, and cognitive decline [36, 53–55, 57–62].

Beyond mobility impairment, slower gait speed has been established as a robust predictor of mortality and late-life cognitive decline, independent of traditional risk factors [57, 61, 63–67]. Longitudinal studies have demonstrated that declining gait speed precedes the onset of dementia and correlates with cortical atrophy, subclinical cerebrovascular pathology, and reduced executive function [53, 58, 63, 64]. The association between gait speed and cognitive decline likely reflects shared neurophysiological pathways, including frontostriatal and corticospinal network deterioration, which compromise both motor control and cognitive processing. Additionally, vascular aging, white matter hyperintensities, and cerebral microinfarcts—all linked to gait slowing—further reinforce the role of gait speed as an early marker of neurovascular dysfunction [14, 15, 19, 20, 24, 39, 40, 68–73]. Interestingly, stride width did not exhibit significant age-related changes, suggesting that postural control adjustments may compensate for other mobility impairments in aging individuals. However, dual-task cost for stride width was significantly associated with age, indicating that cognitive load affects lateral stability more in older adults. This may reflect an increased reliance on postural control mechanisms to maintain balance under cognitively demanding conditions.

Given its predictive value, gait speed measurement, along with other age-sensitive gait parameters, will be integrated into routine functional assessments within the Semmelweis Study to identify individuals at risk for accelerated aging, mobility decline, and cognitive impairment [7, 8]. Tracking longitudinal changes in gait speed across a large, well-characterized workplace cohort will enable the integration of gait metrics into physiology-based biological age indices, providing crucial insights into aging trajectories. This approach could enhance our understanding of the determinants of unhealthy aging, inform preventive strategies, and guide the development of personalized interventions aimed at preserving cognitive and motor function.

Gait asymmetry has been recognized as a sensitive marker of brain health and cognitive dysfunction, and our findings provide further evidence supporting its relevance in aging [25, 29–31, 39]. Among spatial gait parameters, step length asymmetry exhibited a strong positive correlation with age across both single- and dual-task conditions, whereas stride length and stride width asymmetries remained largely unaffected. Similarly, asymmetry in temporal gait parameters and gait cycle components did not demonstrate consistent age-related effects, suggesting that step length asymmetry may be the most relevant indicator of functional decline in aging populations. This pattern likely reflects subtle impairments in neuromuscular coordination and sensorimotor processing, which may precede more overt mobility limitations. Notably, dual-task cost did not correlate with age for gait cycle parameters, indicating that asymmetry in basic gait cycle components develops independently of cognitive load.

Dual-task walking is widely used to assess cognitive-motor interference, and our results confirm that cognitive load exacerbates gait alterations, particularly in older adults [39, 41, 74]. As expected, dual-task conditions significantly affected gait speed, cadence, and stride length in both age groups, reflecting the expected cost of divided attention [39, 41]. However, dual-task cost did not correlate with age for many other gait parameters, suggesting that the impact of cognitive load on gait may not be universally age-dependent but rather influenced by individual variability in cognitive-motor integration and reserve.

These findings provide important insights for the gait analysis strategy of the Semmelweis Study, emphasizing the need to evaluate both single- and dual-task gait conditions to differentiate age-related changes from cognitive load effects. The fact that certain gait parameters remain relatively unaffected by dual-tasking suggests that a targeted selection of gait metrics, particularly those linked to asymmetry and variability, may offer the most sensitive indicators of cognitive-motor interactions in aging populations. Integrating dual-task gait assessments into the Semmelweis Study’s longitudinal framework will allow for early identification of individuals at risk for cognitive decline and functional impairment [39, 41], further reinforcing the study’s role in advancing preventive strategies for healthy aging. Our findings align with preclinical and clinical studies linking gait asymmetry to underlying cerebrovascular pathology, including white matter hyperintensities, cerebral microbleeds, and clinical and subclinical stroke lesions [26, 27, 29, 31, 39, 40]. These structural brain alterations may contribute to impaired interhemispheric communication and reduced neuromotor control, resulting in greater limb-to-limb variability in gait mechanics. Given the established connection between vascular aging and cognitive impairment [75–82], our results further support the use of gait asymmetry as an early indicator of neurovascular dysfunction. Integrating gait asymmetry assessments into longitudinal studies, such as the Semmelweis Study, could enhance early detection of cerebrovascular abnormalities and aid in the identification of individuals at risk for functional decline and cognitive deterioration. Tracking asymmetry patterns over time may help distinguish transient compensatory changes from progressive neurovascular dysfunction, enabling timely interventions to mitigate mobility decline and cognitive impairment. Additionally, by integrating gait analysis with vascular, metabolic, and neurocognitive assessments [16], the Semmelweis Study can provide a more comprehensive understanding of how systemic aging processes influence mobility and cognitive health. This approach could help identify modifiable risk factors, paving the way for targeted interventions, workplace health programs, and lifestyle modifications aimed at preserving functional independence in aging populations.

Several limitations should be considered when interpreting the findings of this pilot study. First, this pilot study employed a cross-sectional design, limiting our ability to infer causal relationships between aging, gait alterations, and cognitive decline. Future longitudinal analyses within the Semmelweis Study will be critical for understanding how gait asymmetry and other gait alterations evolve over time and whether they can reliably predict functional and cognitive decline. Second, our study focused on spatiotemporal gait parameters, but additional kinematic and kinetic analyses may provide deeper mechanistic insights into the origins of gait asymmetry in aging. Incorporating neuroimaging techniques to assess brain structural and functional correlates of gait asymmetry could further elucidate the neural mechanisms underlying age-related gait changes.

In conclusion, this study highlights the importance of gait asymmetry and cognitive load effects as key indicators of age-related decline in brain health, reinforcing their relevance for aging research and clinical assessments. Our findings demonstrate that step length asymmetry serves as a robust marker of functional decline in aging individuals. Additionally, the strong association between gait speed and aging aligns with its role as a predictor of frailty, cognitive impairment, and mortality [7, 8, 57, 83], further emphasizing the need for objective gait assessments in the Semmelweis Study. As part of the Semmelweis Study framework, this pilot investigation establishes a standardized methodology for high-throughput gait analysis, ensuring its integration into longitudinal assessments of neurovascular and cognitive health. By incorporating dual-task gait metrics and asymmetry indices, this approach enables the early identification of individuals at risk for functional and cognitive decline, providing a foundation for targeted interventions to support healthy aging. Future research within the Semmelweis Study will focus on tracking gait asymmetry patterns over time, evaluating their predictive value for neurodegenerative and cerebrovascular changes, and integrating multimodal approaches—including neuroimaging, metabolic profiling, and vascular assessments—to develop a comprehensive framework for understanding mobility and cognitive aging. Additionally, there is a need to expand the Semmelweis Study gait analysis pipeline by incorporating novel gait metrics such as gait variability [18, 32, 84–87] and entropy s [20, 88–94], which provide deeper insights into locomotor control, adaptability, and neuromuscular stability in aging populations. These insights not only enhance our understanding of age-related gait changes but also inform workplace health promotion programs, fall prevention strategies, and personalized intervention models.

## Data Availability

The datasets generated and analyzed during the current study are not publicly available due to privacy restrictions. Anonymized data are available from the corresponding author upon reasonable request and will be made accessible in accordance with institutional and ethical guidelines.

## References

[1] Eurostat. Ageing Europe. LOOKING AT THE LIVES OF OLDER PEOPLE IN THE EU. 2020 edition. https://ec.europa.eu/eurostat/documents/3217494/11478057/KS-02-20-655-EN-N.pdf/9b09606c-d4e8-4c33-63d2-3b20d5c19c91?t=1604055531000 (accessed on 05/16/2021).

[2] Eurostat: Aging Europe. https://ec.europa.eu/eurostat/cache/digpub/ageing/ (accessed on 11/04/2022).

[3] Hungarian Central Statistics Office. STADAT tables 22.1.1.4. https://www.ksh.hu/stadat_files/nep/hu/nep0004.html (accessed on 05/05/2022).

[4] European Commission: The 2015 Aging Report. Underlying assumptions and projection methodologies. European Economy 8/2014 The 2015 aging report: underlying assumptions and projection methodologies (europa.eu) (accessed on 05/05/2022). In.

[5] Ungvari Z, Adany R, Szabo AJ, Dornyei G, Moizs M, Purebl G, Kalabay L, Varga P, Torzsa P, Kellermayer M, Merkely B. Semmelweis Caring University Model Program based on the development of a center of preventive services: health for all employees at a university occupational setting. Front Public Health. 2021;9:727668. 10.3389/fpubh.2021.727668. 34912767 10.3389/fpubh.2021.727668 PMC8666717

[6] Ungvari Z, Tabak AG, Adany R, Purebl G, Kaposvari C, Fazekas-Pongor V, Csipo T, Szarvas Z, Horvath K, Mukli P, Balog P, Bodizs R, Ujma P, Stauder A, Belsky DW, Kovacs I, Yabluchanskiy A, Maier AB, Moizs M, Ostlin P, Yon Y, Varga P, Voko Z, Papp M, Takacs I, Vasarhelyi B, Torzsa P, Ferdinandy P, Csiszar A, Benyo Z, et al. The Semmelweis Study: a longitudinal occupational cohort study within the framework of the Semmelweis Caring University Model Program for supporting healthy aging. Geroscience. 2023. 10.1007/s11357-023-01018-7. 38060158 10.1007/s11357-023-01018-7 PMC10828351

[7] Studenski S, Perera S, Patel K, Rosano C, Faulkner K, Inzitari M, Brach J, Chandler J, Cawthon P, Connor EB, Nevitt M, Visser M, Kritchevsky S, Badinelli S, Harris T, Newman AB, Cauley J, Ferrucci L, Guralnik J. Gait speed and survival in older adults. JAMA. 2011;305:50–8. 10.1001/jama.2010.1923. 21205966 10.1001/jama.2010.1923 PMC3080184

[8] Rosano C, Studenski SA, Aizenstein HJ, Boudreau RM, Longstreth WT Jr, Newman AB. Slower gait, slower information processing and smaller prefrontal area in older adults. Age Ageing. 2012;41:58–64. 10.1093/ageing/afr113. 21965414 10.1093/ageing/afr113 PMC3234076

[9] Rosso AL, Studenski SA, Chen WG, Aizenstein HJ, Alexander NB, Bennett DA, Black SE, Camicioli R, Carlson MC, Ferrucci L, Guralnik JM, Hausdorff JM, Kaye J, Launer LJ, Lipsitz LA, Verghese J, Rosano C. Aging, the central nervous system, and mobility. J Gerontol A Biol Sci Med Sci. 2013;68:1379–86. 10.1093/gerona/glt089. 23843270 10.1093/gerona/glt089 PMC3805295

[10] Rosano C, Rosso AL, Studenski SA. Aging, brain, and mobility: progresses and opportunities. J Gerontol A Biol Sci Med Sci. 2014;69:1373–4. 10.1093/gerona/glu159. 25199913 10.1093/gerona/glu159

[11] Verghese J, Robbins M, Holtzer R, Zimmerman M, Wang C, Xue X, Lipton RB. Gait dysfunction in mild cognitive impairment syndromes. J Am Geriatr Soc. 2008;56:1244–51. 10.1111/j.1532-5415.2008.01758.x. 18482293 10.1111/j.1532-5415.2008.01758.x PMC2574944

[12] Holtzer R, Verghese J, Xue X, Lipton RB. Cognitive processes related to gait velocity: results from the Einstein Aging Study. Neuropsychology. 2006;20:215–23. 10.1037/0894-4105.20.2.215. 16594782 10.1037/0894-4105.20.2.215

[13] Oveisgharan S, Yu L, Wang T, Schneider JA, Bennett DA, Buchman AS. Neurodegenerative and cerebrovascular brain pathologies are differentially associated with declining grip strength and gait in older adults. J Gerontol A Biol Sci Med Sci. 2023;78:504–13. 10.1093/gerona/glac128. 35675284 10.1093/gerona/glac128 PMC9977235

[14] Ungvari Z, Tarantini S, Hertelendy P, Valcarcel-Ares MN, Fulop GA, Logan S, Kiss T, Farkas E, Csiszar A, Yabluchanskiy A. Cerebromicrovascular dysfunction predicts cognitive decline and gait abnormalities in a mouse model of whole brain irradiation-induced accelerated brain senescence. Geroscience. 2017;39:33–42. 10.1007/s11357-017-9964-z. 28299642 10.1007/s11357-017-9964-z PMC5352588

[15] Tarantini S, Yabluchanksiy A, Fulop GA, Hertelendy P, Valcarcel-Ares MN, Kiss T, Bagwell JM, O’Connor D, Farkas E, Sorond F, Csiszar A, Ungvari Z. Pharmacologically induced impairment of neurovascular coupling responses alters gait coordination in mice. Geroscience. 2017;39:601–14. 10.1007/s11357-017-0003-x. 29243191 10.1007/s11357-017-0003-x PMC5745218

[16] Sorond FA, Galica A, Serrador JM, Kiely DK, Iloputaife I, Cupples LA, Lipsitz LA. Cerebrovascular hemodynamics, gait, and falls in an elderly population: MOBILIZE Boston Study. Neurology. 2010;74:1627–33. 10.1212/WNL.0b013e3181df0982. 20479362 10.1212/WNL.0b013e3181df0982 PMC2875129

[17] Rosano C, Longstreth WT Jr, Boudreau R, Taylor CA, Du Y, Kuller LH, Newman AB. High blood pressure accelerates gait slowing in well-functioning older adults over 18-years of follow-up. J Am Geriatr Soc. 2011;59:390–7. 10.1111/j.1532-5415.2010.03282.x. 21391929 10.1111/j.1532-5415.2010.03282.x PMC3637929

[18] Rosano C, Brach J, Studenski S, Longstreth WT Jr, Newman AB. Gait variability is associated with subclinical brain vascular abnormalities in high-functioning older adults. Neuroepidemiology. 2007;29:193–200. 10.1159/000111582. 18043004 10.1159/000111582 PMC2824582

[19] Pinter D, Ritchie SJ, Doubal F, Gattringer T, Morris Z, Bastin ME, Del CVHM, Royle NA, Corley J, Munoz Maniega S, Pattie A, Dickie DA, Staals J, Gow AJ, Starr JM, Deary IJ, Enzinger C, Fazekas F, Wardlaw J. Impact of small vessel disease in the brain on gait and balance. Sci Rep. 2017;7:41637. 10.1038/srep41637. 28134332 10.1038/srep41637 PMC5278543

[20] Mukli P, Detwiler S, Owens CD, Csipo T, Lipecz A, Pinto CB, Tarantini S, Nyul-Toth A, Balasubramanian P, Hoffmeister JR, Csiszar A, Ungvari Z, Kirkpatrick AC, Prodan CI, Yabluchanskiy A. Gait variability predicts cognitive impairment in older adults with subclinical cerebral small vessel disease. Front Aging Neurosci. 2022;14:1052451. 10.3389/fnagi.2022.1052451. 36466602 10.3389/fnagi.2022.1052451 PMC9716182

[21] Hollman JH, McDade EM, Petersen RC. Normative spatiotemporal gait parameters in older adults. Gait Posture. 2011;34:111–8. 10.1016/j.gaitpost.2011.03.024. 21531139 10.1016/j.gaitpost.2011.03.024 PMC3104090

[22] Savica R, Wennberg AM, Hagen C, Edwards K, Roberts RO, Hollman JH, Knopman DS, Boeve BF, Machulda MM, Petersen RC, Mielke MM. Comparison of gait parameters for predicting cognitive decline: the Mayo clinic study of aging. J Alzheimers Dis. 2017;55:559–67. 10.3233/JAD-160697. 27662317 10.3233/JAD-160697 PMC5378311

[23] Wennberg AMV, Lesnick TG, Schwarz CG, Savica R, Hagen CE, Roberts RO, Knopman DS, Hollman JH, Vemuri P, Jack CR Jr, Petersen RC, Mielke MM. Longitudinal Association Between Brain Amyloid-Beta and Gait in the Mayo Clinic Study of Aging. J Gerontol A Biol Sci Med Sci. 2018;73:1244–50. 10.1093/gerona/glx240. 29236984 10.1093/gerona/glx240 PMC6093355

[24] Nyul-Toth A, Tarantini S, Kiss T, Toth P, Galvan V, Tarantini A, Yabluchanskiy A, Csiszar A, Ungvari Z. Increases in hypertension-induced cerebral microhemorrhages exacerbate gait dysfunction in a mouse model of Alzheimer’s disease. Geroscience. 2020. 10.1007/s11357-020-00256-3. 32844283 10.1007/s11357-020-00256-3 PMC7732885

[25] Peng D, Zhou Y, Diao Y, Chen G, Wang Y, Ning Y, Li G, Wang W, Zhao G. Quantitative analysis of the gait variability and asymmetry using inertial measurement unit during dual-task gait. Annu Int Conf IEEE Eng Med Biol Soc. 2024;2024:1–6. 10.1109/EMBC53108.2024.10782256. 40039228 10.1109/EMBC53108.2024.10782256

[26] Szopa A, Domagalska-Szopa M, Lasek-Bal A, Zak A. The link between weight shift asymmetry and gait disturbances in chronic hemiparetic stroke patients. Clin Interv Aging. 2017;12:2055–62. 10.2147/CIA.S144795. 29238181 10.2147/CIA.S144795 PMC5716326

[27] Alexander LD, Black SE, Patterson KK, Gao F, Danells CJ, McIlroy WE. Association between gait asymmetry and brain lesion location in stroke patients. Stroke. 2009;40:537–44. 10.1161/STROKEAHA.108.527374. 19109546 10.1161/STROKEAHA.108.527374

[28] Ichihashi N, Ikezoe T, Sato S, Ibuki S. Gait asymmetry assessment for older adults by measuring circular gait speed. Geriatr Gerontol Int. 2019;19:736–9. 10.1111/ggi.13691. 31106945 10.1111/ggi.13691

[29] Nagano H, Sarashina E, Sparrow W, Mizukami K, Begg R. General mental health is associated with gait asymmetry. Sensors (Basel). 2019;19. 10.3390/s19224908 10.3390/s19224908 PMC6891551 31717634

[30] Orcioli-Silva D, Barbieri FA, Dos Santos PCR, Beretta VS, Simieli L, Vitorio R, Lirani-Silva E, Gobbi LTB. Double obstacles increase gait asymmetry during obstacle crossing in people with Parkinson’s disease and healthy older adults: a pilot study. Sci Rep. 2020;10:2272. 10.1038/s41598-020-59266-y. 32042027 10.1038/s41598-020-59266-y PMC7010667

[31] Zadik S, Benady A, Gutwillig S, Florentine MM, Solymani RE, Plotnik M. Age related changes in gait variability, asymmetry, and bilateral coordination-when does deterioration starts? Gait Posture. 2022;96:87–92. 10.1016/j.gaitpost.2022.05.009. 35617787 10.1016/j.gaitpost.2022.05.009

[32] Brach JS, Perera S, Studenski S, Katz M, Hall C, Verghese J. Meaningful change in measures of gait variability in older adults. Gait Posture. 2010;31:175–9. 10.1016/j.gaitpost.2009.10.002. 19889543 10.1016/j.gaitpost.2009.10.002 PMC2818277

[33] Callisaya ML, Blizzard L, Schmidt MD, McGinley JL, Srikanth VK. Ageing and gait variability–a population-based study of older people. Age Ageing. 2010;39:191–7. 10.1093/ageing/afp250. 20083617 10.1093/ageing/afp250

[34] Beauchet O, Launay C, Annweiler C, Fantino B, Allali G, De Decker L. Physical training-related changes in gait variability while single and dual tasking in older adults: magnitude of gait variability at baseline matters. Eur J Phys Rehabil Med. 2013;49:857–64. 24285023

[35] Wittwer JE, Webster KE, Hill K. Reproducibility of gait variability measures in people with Alzheimer’s disease. Gait Posture. 2013;38:507–10. 10.1016/j.gaitpost.2013.01.021. 23485356 10.1016/j.gaitpost.2013.01.021

[36] Gillain S, Drame M, Lekeu F, Wojtasik V, Ricour C, Croisier JL, Salmon E, Petermans J. Gait speed or gait variability, which one to use as a marker of risk to develop Alzheimer disease? A pilot study. Aging Clin Exp Res. 2016;28:249–55. 10.1007/s40520-015-0392-6. 26076908 10.1007/s40520-015-0392-6

[37] Beauchet O, Launay CP, Sekhon H, Barthelemy JC, Roche F, Chabot J, Levinoff EJ, Allali G. Association of increased gait variability while dual tasking and cognitive decline: results from a prospective longitudinal cohort pilot study. Geroscience. 2017. 10.1007/s11357-017-9992-8. 28825181 10.1007/s11357-017-9992-8 PMC5636771

[38] Pieruccini-Faria F, Black SE, Masellis M, Smith EE, Almeida QJ, Li KZH, Bherer L, Camicioli R, Montero-Odasso M. Gait variability across neurodegenerative and cognitive disorders: results from the Canadian Consortium of Neurodegeneration in Aging (CCNA) and the Gait and Brain Study. Alzheimers Dement. 2021. 10.1002/alz.12298. 33590967 10.1002/alz.12298 PMC8451764

[39] Xie H, Xia C, Zhao H, Xia Z, Zhang N, Huang Y. Variability, asymmetry and bilateral coordination of gait during single- and dual-task walking of patients with cerebral small vessel disease. Int J Neurosci. 2024:1–10. 10.1080/00207454.2024.2309454 10.1080/00207454.2024.2309454 38294519

[40] Ungvari Z, Muranyi M, Gulej R, Negri S, Nyul-Toth A, Csik B, Patai R, Conley S, Milan M, Bagwell J, O’Connor D, Tarantini A, Yabluchanskiy A, Toth P, Csiszar A, Ungvari A, Mukli P, Tarantini S. Longitudinal detection of gait alterations associated with hypertension-induced cerebral microhemorrhages in mice: predictive role of stride length and stride time asymmetry and increased gait entropy. Geroscience. 2024;46:4743–60. 10.1007/s11357-024-01210-3. 38914916 10.1007/s11357-024-01210-3 PMC11335995

[41] Hollman JH, Kovash FM, Kubik JJ, Linbo RA. Age-related differences in spatiotemporal markers of gait stability during dual task walking. Gait Posture. 2007;26:113–9. 10.1016/j.gaitpost.2006.08.005. 16959488 10.1016/j.gaitpost.2006.08.005

[42] Hollman JH, Youdas JW, Lanzino DJ. Gender differences in dual task gait performance in older adults. Am J Mens Health. 2011;5:11–7. 10.1177/1557988309357232. 20031935 10.1177/1557988309357232

[43] Ayers EI, Tow AC, Holtzer R, Verghese J. Walking while talking and falls in aging. Gerontology. 2014;60:108–13. 10.1159/000355119. 24192342 10.1159/000355119 PMC3944080

[44] Montero-Odasso M, Oteng-Amoako A, Speechley M, Gopaul K, Beauchet O, Annweiler C, Muir-Hunter SW. The motor signature of mild cognitive impairment: results from the gait and brain study. J Gerontol A Biol Sci Med Sci. 2014;69:1415–21. 10.1093/gerona/glu155. 25182601 10.1093/gerona/glu155 PMC4197903

[45] Montero-Odasso M, Verghese J, Beauchet O, Hausdorff JM. Gait and cognition: a complementary approach to understanding brain function and the risk of falling. J Am Geriatr Soc. 2012;60:2127–36. 10.1111/j.1532-5415.2012.04209.x. 23110433 10.1111/j.1532-5415.2012.04209.x PMC3498517

[46] Modarresi S, Divine A, Grahn JA, Overend TJ, Hunter SW. Gait parameters and characteristics associated with increased risk of falls in people with dementia: a systematic review. Int Psychogeriatr. 2019;31:1287–303. 10.1017/S1041610218001783. 30520404 10.1017/S1041610218001783

[47] Donoghue OA, Leahy S, Kenny RA. Longitudinal associations between gait, falls, and disability in community-dwelling older adults with type II diabetes mellitus: findings from the Irish longitudinal study on ageing (TILDA). J Gerontol A Biol Sci Med Sci. 2021;76:906–13. 10.1093/gerona/glaa263. 33049045 10.1093/gerona/glaa263

[48] Blackwood J, Rybicki K. Assessment of gait speed and timed up and go measures as predictors of falls in older breast cancer survivors. Integr Cancer Ther. 2021;20:15347354211006462. 10.1177/15347354211006462. 33784836 10.1177/15347354211006462 PMC8020039

[49] Beck Jepsen D, Robinson K, Ogliari G, Montero-Odasso M, Kamkar N, Ryg J, Freiberger E, Masud T. Predicting falls in older adults: an umbrella review of instruments assessing gait, balance, and functional mobility. BMC Geriatr. 2022;22:615. 10.1186/s12877-022-03271-5. 35879666 10.1186/s12877-022-03271-5 PMC9310405

[50] Viteckova S, Kutilek P, Svoboda Z, Krupicka R, Kauler J, Szabo Z. Gait symmetry measures: a review of current and prospective methods. Biomed Signal Process Control. 2018;42:89–100. 10.1016/j.bspc.2018.01.013.

[51] Ungvari Z, Tabak AG, Adany R, Purebl G, Kaposvari C, Fazekas-Pongor V, Csipo T, Szarvas Z, Horvath K, Mukli P, Balog P, Bodizs R, Ujma P, Stauder A, Belsky DW, Kovacs I, Yabluchanskiy A, Maier AB, Moizs M, Ostlin P, Yon Y, Varga P, Voko Z, Papp M, Takacs I, Vasarhelyi B, Torzsa P, Ferdinandy P, Csiszar A, Benyo Z, et al. The Semmelweis Study: a longitudinal occupational cohort study within the framework of the Semmelweis Caring University Model Program for supporting healthy aging. Geroscience. 2024;46:191–218. 10.1007/s11357-023-01018-7. 38060158 10.1007/s11357-023-01018-7 PMC10828351

[52] Oh-Park M, Holtzer R, Xue X, Verghese J. Conventional and robust quantitative gait norms in community-dwelling older adults. J Am Geriatr Soc. 2010;58:1512–8. 10.1111/j.1532-5415.2010.02962.x. 20646103 10.1111/j.1532-5415.2010.02962.x PMC2955162

[53] Watson NL, Rosano C, Boudreau RM, Simonsick EM, Ferrucci L, Sutton-Tyrrell K, Hardy SE, Atkinson HH, Yaffe K, Satterfield S, Harris TB, Newman AB. Executive function, memory, and gait speed decline in well-functioning older adults. J Gerontol A Biol Sci Med Sci. 2010;65:1093–100. 10.1093/gerona/glq111. 20581339 10.1093/gerona/glq111 PMC2949334

[54] Rosso AL, Sanders JL, Arnold AM, Boudreau RM, Hirsch CH, Carlson MC, Rosano C, Kritchevsky SB, Newman AB. Multisystem physiologic impairments and changes in gait speed of older adults. J Gerontol A Biol Sci Med Sci. 2015;70:319–24. 10.1093/gerona/glu176. 25380599 10.1093/gerona/glu176 PMC4351395

[55] Fitzpatrick AL, Buchanan CK, Nahin RL, Dekosky ST, Atkinson HH, Carlson MC, Williamson JD. Associations of gait speed and other measures of physical function with cognition in a healthy cohort of elderly persons. J Gerontol A Biol Sci Med Sci. 2007;62:1244–51. 18000144 10.1093/gerona/62.11.1244

[56] Sprague BN, Zhu X, Rosso AL, VerghesDelbaereLipnickiSachdevNgGweeYapKimHanOhNarazakiChenChenBrodatyNumbersKochanWalkerPaddickGurejeOjagbemiBelloRosano JKDMPSTPXKBKWJWDJKTSHXNARWSMOATC, Consortium C. Correlates of gait speed among older adults from 6 countries: findings from the COSMIC collaboration. J Gerontol A Biol Sci Med Sci. 2023;78:2396–406. 10.1093/gerona/glad090. 36975099 10.1093/gerona/glad090 PMC10692426

[57] Perera S, Patel KV, Rosano C, Rubin SM, Satterfield S, Harris T, Ensrud K, Orwoll E, Lee CG, Chandler JM, Newman AB, Cauley JA, Guralnik JM, Ferrucci L, Studenski SA. Gait speed predicts incident disability: a pooled analysis. J Gerontol A Biol Sci Med Sci. 2015. 10.1093/gerona/glv126. 26297942 10.1093/gerona/glv126 PMC4715231

[58] Peel NM, Alapatt LJ, Jones LV, Hubbard RE. The association between gait speed and cognitive status in community-dwelling older people: a systematic review and meta-analysis. J Gerontol A Biol Sci Med Sci. 2019;74:943–8. 10.1093/gerona/gly140. 29917045 10.1093/gerona/gly140

[59] Inzitari M, Newman AB, Yaffe K, Boudreau R, de Rekeneire N, Shorr R, Harris TB, Rosano C. Gait speed predicts decline in attention and psychomotor speed in older adults: the health aging and body composition study. Neuroepidemiology. 2007;29:156–62. 10.1159/000111577. 18042999 10.1159/000111577 PMC2824580

[60] Atkinson HH, Rosano C, Simonsick EM, Williamson JD, Davis C, Ambrosius WT, Rapp SR, Cesari M, Newman AB, Harris TB, Rubin SM, Yaffe K, Satterfield S, Kritchevsky SB. Cognitive function, gait speed decline, and comorbidities: the health, aging and body composition study. J Gerontol A Biol Sci Med Sci. 2007;62:844–50. 17702875 10.1093/gerona/62.8.844

[61] Abellan van Kan G, Rolland Y, Andrieu S, Bauer J, Beauchet O, Bonnefoy M, Cesari M, Donini LM, Gillette Guyonnet S, Inzitari M, Nourhashemi F, Onder G, Ritz P, Salva A, Visser M, Vellas B. Gait speed at usual pace as a predictor of adverse outcomes in community-dwelling older people an International Academy on Nutrition and Aging (IANA) Task Force. J Nutr Health Aging. 2009;13:881–889.10.1007/s12603-009-0246-z19924348

[62] Verghese J, Holtzer R, Lipton RB, Wang C. Quantitative gait markers and incident fall risk in older adults. J Gerontol A Biol Sci Med Sci. 2009;64:896–901. 10.1093/gerona/glp033. 19349593 10.1093/gerona/glp033 PMC2709543

[63] White DK, Neogi T, Nevitt MC, Peloquin CE, Zhu Y, Boudreau RM, Cauley JA, Ferrucci L, Harris TB, Satterfield SM, Simonsick EM, Strotmeyer ES, Zhang Y. Trajectories of gait speed predict mortality in well-functioning older adults: the health, aging and body composition study. J Gerontol A Biol Sci Med Sci. 2013;68:456–64. 10.1093/gerona/gls197. 23051974 10.1093/gerona/gls197 PMC3593620

[64] Jung HW, Jang IY, Lee CK, Yu SS, Hwang JK, Jeon C, Lee YS, Lee E. Usual gait speed is associated with frailty status, institutionalization, and mortality in community-dwelling rural older adults: a longitudinal analysis of the Aging Study of Pyeongchang Rural Area. Clin Interv Aging. 2018;13:1079–89. 10.2147/CIA.S166863. 29922046 10.2147/CIA.S166863 PMC5995421

[65] Veronese N, Stubbs B, Volpato S, Zuliani G, Maggi S, Cesari M, Lipnicki DM, Smith L, Schofield P, Firth J, Vancampfort D, Koyanagi A, Pilotto A, Cereda E. Association between gait speed with mortality, cardiovascular disease and cancer: a systematic review and meta-analysis of prospective cohort studies. J Am Med Dir Assoc. 2018;19(981–988):e987. 10.1016/j.jamda.2018.06.007. 10.1016/j.jamda.2018.06.007 30056008

[66] Dommershuijsen LJ, Isik BM, Darweesh SKL, van der Geest JN, Ikram MK, Ikram MA. Unraveling the association between gait and mortality-one step at a time. J Gerontol A Biol Sci Med Sci. 2020;75:1184–90. 10.1093/gerona/glz282. 31807749 10.1093/gerona/glz282 PMC7243583

[67] Doi T, Nakakubo S, Tsutsumimoto K, Kurita S, Ishii H, Shimada H. Spatiotemporal gait characteristics and risk of mortality in community-dwelling older adults. Maturitas. 2021;151:31–35. 10.1016/j.maturitas.2021.06.007 10.1016/j.maturitas.2021.06.007 34446276

[68] van der Holst HM, Tuladhar AM, Zerbi V, van Uden IWM, de Laat KF, van Leijsen EMC, Ghafoorian M, Platel B, Bergkamp MI, van Norden AGW, Norris DG, van Dijk EJ, Kiliaan AJ, de Leeuw FE. White matter changes and gait decline in cerebral small vessel disease. Neuroimage Clin. 2018;17:731–8. 10.1016/j.nicl.2017.12.007. 29270357 10.1016/j.nicl.2017.12.007 PMC5730123

[69] de Laat KF, Tuladhar AM, van Norden AG, Norris DG, Zwiers MP, de Leeuw FE. Loss of white matter integrity is associated with gait disorders in cerebral small vessel disease. Brain. 2011;134:73–83. 10.1093/brain/awq343. 21156660 10.1093/brain/awq343

[70] Sharma B, Wang M, McCreary CR, Camicioli R, Smith EE. Gait and falls in cerebral small vessel disease: a systematic review and meta-analysis. Age Ageing. 2023;52. 10.1093/ageing/afad011 10.1093/ageing/afad011 PMC10064981 37000039

[71] Siejka TP, Srikanth VK, Hubbard RE, Moran C, Beare R, Wood A, Phan T, Balogun S, Callisaya ML. White matter hyperintensities and the progression of frailty-the Tasmanian Study of cognition and gait. J Gerontol A Biol Sci Med Sci. 2020;75:1545–50. 10.1093/gerona/glaa024. 31956917 10.1093/gerona/glaa024

[72] Nadkarni NK, Nunley KA, Aizenstein H, Harris TB, Yaffe K, Satterfield S, Newman AB, Rosano C. Association between cerebellar gray matter volumes, gait speed, and information-processing ability in older adults enrolled in the Health ABC study. J Gerontol A Biol Sci Med Sci. 2014;69:996–1003. 10.1093/gerona/glt151. 24170673 10.1093/gerona/glt151 PMC4095927

[73] de Laat KF, van den Berg HA, van Norden AG, Gons RA, Olde Rikkert MG, de Leeuw FE. Microbleeds are independently related to gait disturbances in elderly individuals with cerebral small vessel disease. Stroke. 2011;42:494–7. 10.1161/STROKEAHA.110.596122. 21164137 10.1161/STROKEAHA.110.596122

[74] Suri A, VanSwearingen J, Rosano C, Brach JS, Redfern MS, Sejdic E, Rosso AL. Uneven surface and cognitive dual-task independently affect gait quality in older adults. Gait Posture. 2023;106:34–41. 10.1016/j.gaitpost.2023.08.010. 37647710 10.1016/j.gaitpost.2023.08.010 PMC10591986

[75] van der Flier WM, Skoog I, Schneider JA, Pantoni L, Mok V, Chen CLH, Scheltens P. Vascular cognitive impairment. Nat Rev Dis Primers. 2018;4:18003. 10.1038/nrdp.2018.3. 29446769 10.1038/nrdp.2018.3

[76] Dichgans M, Leys D. Vascular Cognitive Impairment. Circ Res. 2017;120:573–91. 10.1161/CIRCRESAHA.116.308426. 28154105 10.1161/CIRCRESAHA.116.308426

[77] Mahinrad S, Sorond F, Gorelick PB. The role of vascular risk factors in cognitive impairment and dementia and prospects for prevention. Clin Geriatr Med. 2023;39:123–34. 10.1016/j.cger.2022.07.007. 36404025 10.1016/j.cger.2022.07.007 PMC11806923

[78] Csipo T, Mukli P, Lipecz A, Tarantini S, Bahadli D, Abdulhussein O, Owens C, Kiss T, Balasubramanian P, Nyul-Toth A, Hand RA, Yabluchanska V, Sorond FA, Csiszar A, Ungvari Z, Yabluchanskiy A. Assessment of age-related decline of neurovascular coupling responses by functional near-infrared spectroscopy (fNIRS) in humans. Geroscience. 2019;41:495–509. 10.1007/s11357-019-00122-x. 31676966 10.1007/s11357-019-00122-x PMC6885078

[79] Iadecola C, Smith EE, Anrather J, Gu C, Mishra A, Misra S, Perez-Pinzon MA, Shih AY, Sorond FA, van Veluw SJ, Wellington CL, American Heart Association Stroke Council Council on Arteriosclerosis T, Vascular Biology Council on Cardiovascular R, Intervention Council on Hypertension Council on L, Cardiometabolic H. The neurovasculome: key roles in brain health and cognitive impairment: a scientific statement from the American Heart Association/American Stroke Association. Stroke. 2023;54:e251-e271. 10.1161/STR.0000000000000431 10.1161/STR.0000000000000431 PMC10228567 37009740

[80] Gorelick PB, Sorond FA. Advancing our knowledge about cerebral small vessel diseases. Lancet Neurol. 2023;22:972–3. 10.1016/S1474-4422(23)00318-6. 37863595 10.1016/S1474-4422(23)00318-6 PMC11805494

[81] Jenkins LM, Kogan A, Malinab M, Ingo C, Sedaghat S, Bryan NR, Yaffe K, Parrish TB, Nemeth AJ, Lloyd-Jones DM, Launer LJ, Wang L, Sorond F. Blood pressure, executive function, and network connectivity in middle-aged adults at risk of dementia in late life. Proc Natl Acad Sci U S A. 2021;118. 10.1073/pnas.2024265118 10.1073/pnas.2024265118 PMC8449402 34493658

[82] Ingo C, Kurian S, Higgins J, Mahinrad S, Jenkins L, Gorelick P, Lloyd-Jones D, Sorond F. Vascular health and diffusion properties of normal appearing white matter in midlife. Brain Commun. 2021;3:fcab080. 10.1093/braincomms/fcab080 10.1093/braincomms/fcab080 PMC8087895 34494002

[83] Bair WN, Petr M, Alfaras I, Mitchell SJ, Bernier M, Ferrucci L, Studenski SA, De Cabo R. Of aging mice and men: gait speed decline is a translatable trait, with species-specific underlying properties. J Gerontol A Biol Sci Med Sci. 2019;74:1413–6. 10.1093/gerona/glz015. 30649206 10.1093/gerona/glz015 PMC6696716

[84] Decker LM, Cignetti F, Hunt N, Potter JF, Stergiou N, Studenski SA. Effects of aging on the relationship between cognitive demand and step variability during dual-task walking. Age (Dordr). 2016;38:363–75. 10.1007/s11357-016-9941-y. 27488838 10.1007/s11357-016-9941-y PMC5061669

[85] Brach JS, Studenski S, Perera S, VanSwearingen JM, Newman AB. Stance time and step width variability have unique contributing impairments in older persons. Gait Posture. 2008;27:431–9. 10.1016/j.gaitpost.2007.05.016. 17632004 10.1016/j.gaitpost.2007.05.016 PMC2276116

[86] Rosso AL, Olson Hunt MJ, Yang M, Brach JS, Harris TB, Newman AB, Satterfield S, Studenski SA, Yaffe K, Aizenstein HJ, Rosano C. Higher step length variability indicates lower gray matter integrity of selected regions in older adults. Gait Posture. 2014;40:225–30. 10.1016/j.gaitpost.2014.03.192. 24792638 10.1016/j.gaitpost.2014.03.192 PMC4071448

[87] Tian Q, Chastan N, Bair WN, Resnick SM, Ferrucci L, Studenski SA. The brain map of gait variability in aging, cognitive impairment and dementia-a systematic review. Neurosci Biobehav Rev. 2017;74:149–62. 10.1016/j.neubiorev.2017.01.020. 28115194 10.1016/j.neubiorev.2017.01.020 PMC5303129

[88] Thammachat K, Songkhla SN, Aniwattanapong D, Suriyaamarit D. Reliability and minimal detectable change of nonlinear analysis measure of postural control in older adults with mild cognitive impairment. Gait Posture. 2024;107:152–4. 10.1016/j.gaitpost.2023.06.004. 37321920 10.1016/j.gaitpost.2023.06.004

[89] Zanin M, Gomez-Andres D, Pulido-Valdeolivas I, Martin-Gonzalo JA, Lopez-Lopez J, Pascual-Pascual SI, Rausell E. Characterizing normal and pathological gait through permutation entropy. Entropy (Basel). 2018;20. 10.3390/e20010077 10.3390/e20010077 PMC7512275 33265160

[90] Yentes JM, Raffalt PC. Entropy analysis in gait research: methodological considerations and recommendations. Ann Biomed Eng. 2021;49:979–90. 10.1007/s10439-020-02616-8. 33560467 10.1007/s10439-020-02616-8 PMC8051436

[91] Raffalt PC, Denton W, Yentes JM. On the choice of multiscale entropy algorithm for quantification of complexity in gait data. Comput Biol Med. 2018;103:93–100. 10.1016/j.compbiomed.2018.10.008. 30343216 10.1016/j.compbiomed.2018.10.008 PMC6957257

[92] McCamley JD, Denton W, Arnold A, Raffalt PC, Yentes JM. On the calculation of sample entropy using continuous and discrete human gait data. Entropy (Basel). 2018;20. 10.3390/e20100764 10.3390/e20100764 PMC6402504 30853788

[93] Huang HP, Hsu CF, Mao YC, Hsu L, Chi S. Gait stability measurement by using average entropy. Entropy (Basel). 2021;23. 10.3390/e23040412 10.3390/e23040412 PMC8067110 33807223

[94] Ahmadi S, Wu C, Sepehri N, Kantikar A, Nankar M, Szturm T. The effects of aging and dual tasking on human gait complexity during treadmill walking: a comparative study using quantized dynamical entropy and sample entropy. J Biomech Eng. 2018;140. 10.1115/1.4037945 10.1115/1.4037945 28975279

